# 4D‐STEM Nanoscale Strain Analysis in van der Waals Materials: Advancing beyond Planar Configurations

**DOI:** 10.1002/smsc.202300249

**Published:** 2024-01-12

**Authors:** Maarten Bolhuis, Sabrya E. van Heijst, Jeroen J. M. Sangers, Sonia Conesa‐Boj

**Affiliations:** ^1^ Kavli Institute of Nanoscience Delft University of Technology 2628 CJ Delft The Netherlands

**Keywords:** electron microscope pixel array detector (EMPAD), four‐dimensional scanning transmission electron microscopy (4D‐STEM), strain mapping, van der Waals materials

## Abstract

Achieving nanoscale strain fields mapping in intricate van der Waals (vdW) nanostructures, like twisted flakes and nanorods, presents several challenges due to their complex geometry, small size, and sensitivity limitations. Understanding these strain fields is pivotal as they significantly influence the optoelectronic properties of vdW materials, playing a crucial role in a plethora of applications ranging from nanoelectronics to nanophotonics. Here, a novel approach for achieving a nanoscale‐resolved mapping of strain fields across entire micron‐sized vdW nanostructures using four‐dimensional (4D) scanning transmission electron microscopy (STEM) imaging equipped with an electron microscope pixel array detector (EMPAD) is presented. This technique extends the capabilities of STEM‐based strain mapping by means of the exit‐wave power cepstrum method incorporating automated peak tracking and *K*‐means clustering algorithms. This approach is validated on two representative vdW nanostructures: a two‐dimensional (2D) MoS_2_ thin twisted flakes and a one‐dimensional (1D) MoO_3_/MoS_2_ nanorod heterostructure. Beyond just vdW materials, the versatile methodology offers broader applicability for strain‐field analysis in various low‐dimensional nanostructured materials. This advances the understanding of the intricate relationship between nanoscale strain patterns and their consequent optoelectronic properties.

## Graphical abstract

This study introduces a four‐dimensional scanning transmission electron microscopy technique for nanoscale strain mapping in van der Waals nanostructures. Employing a pixelated detector and advanced computational methods, our approach effectively maps strain fields in geometries like twisted flakes and nanorods. We validate it on MoS_2_ thin flakes and MoO_3_/MoS_2_ nanorod heterostructures, enhancing understanding of strain‐induced optoelectronic properties in low‐dimensional materials.

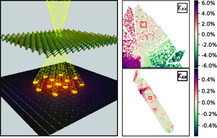

## 1 Introduction

The unique mechanical and electronic properties of two‐dimensional (2D) van der Waals (vdW) materials have made them increasingly relevant in a wide range of fields, from nanoelectronics to nanophotonics. The presence of local strain fields provides a key handle on the functionalities of vdW materials, which is known to affect the resulting optical, electrical, magnetic, and mechanical properties.^[^ ^1^, ^2^, ^3^, ^4^, ^5^, ^6^ ^]^ Consequently, understanding the quantitative implications of local strain fields for the optoelectronic properties of vdW materials is essential for their practical applications.

Transmission electron microscopy (TEM) represents a powerful tool for studying crystal structures with high spatial resolution and benefits from the capability of accessing the underlying strain fields by means of electron diffraction methods. Commonly used methods are selected area diffraction (SAD),^[^ ^7^, ^8^ ^]^ convergent beam electron diffraction (CBED),^[^ ^9^, ^10^ ^]^ and geometric phase analysis (GPA).^[^ ^11^, ^12^, ^13^, ^14^ ^]^ However, implementing these techniques for 2D vdW materials is challenging because they rely on diffraction contrast. The thin layers in a 2D vdW material often produce a weak diffraction signal, making it difficult to accurately quantify the strain fields with the sought‐for nanometer‐scale resolution without the use of specialized patterned probes.^[^ ^15^, ^16^ ^]^ Furthermore, GPA and related methods are fundamentally limited to a small field of view, which prevents comprehensive inspection of large specimens.

In recent years, four‐dimensional (4D) scanning transmission electron microscopy (STEM) has emerged as a promising complementary technique for strain mapping in nanomaterials. 4D‐STEM strain mapping relies on capturing a nanobeam electron diffraction (NBED)^[^ ^17^, ^18^ ^]^ pattern for each electron probe position while scanning the entire specimen, which one can accomplish using specialized detectors like the electron microscope pixel array detector (EMPAD).^[^ ^19^ ^]^ This STEM‐based approach enables the mapping of strain fields across complete, micron‐sized specimens without sacrificing nanoscale resolution by tracking the position of the NBED diffraction disk.^[^ ^18^, ^20^, ^21^, ^22^, ^23^ ^]^ However, achieving nanometer spatial resolution in strain mapping remains challenging for 2D vdW materials configurations beyond the traditional regular flakes. The main difficulty in STEM‐based strain mapping arises for complex nanostructures, such as twisted 2D layers or one‐dimensional (1D) heterostructures. The diffraction patterns from these geometries are challenging to classify, as they contain contributions from different crystal structures, crystal orientations, and materials. In this context, various machine learning methods have been employed to analyze materials at the atomic scale, including deep learning^[^ ^24^ ^]^ and unsupervised clustering algorithms.^[^ ^25^ ^]^ While unsupervised clustering algorithms can classify different deformations in nanomaterials based on similarities in their diffraction patterns, they might fall short in accurately isolating strain deformation in the crystal lattice, especially when strain and deformation intricacies are closely intertwined. Indeed, extracting local strain in complex nanostructures requires disentangling the individual contributions of the different crystal structures and materials in the recorded diffraction pattern. This includes accounting for the contributions from non‐ideal sample tilt. Advanced techniques like precession electron diffraction (PED) can counteract the contributions from non‐ideal sample orientations but require specialized equipment and increase acquisition time.^[^ ^26^, ^27^ ^]^ Additionally, these 2D vdW configurations often exhibit Moiré patterns, further complicating the interpretation of the acquired diffraction patterns regarding strain fields. While recent advances in Bragg interferometry have made it possible to probe strain deformations in twisted graphene and transition metal dichalcogenide (TMD)bilayers,^[^ ^28^, ^29^, ^30^ ^]^ it is still limited to very thin specimens because Bragg interferometry relies on the weak phase approximation to be applicable. The aforementioned limitations highlight the need for general, precise and accurate methods in analyzing 2D vdW materials beyond the planar configuration, especially in cases where the strain and deformation are intertwined.

Here, we tackle all these challenges by extending STEM‐based strain mapping methods by means of the exit‐wave power cepstrum (EWPC) approach.^[^ ^31^ ^]^ The EWPC method has recently been instrumental in strain and grain mapping for a variety of applications, from core–shell nanoparticles to superconducting materials.^[^ ^32^, ^33^, ^34^, ^35^, ^36^ ^]^ Building upon the EWPC approach, we implement automated peak tracking and *K*‐means clustering algorithms to efficiently calculate interatomic distances at various positions across micron‐sized specimens. To demonstrate the reliability of our approach, we apply it to two distinct vdW nanostructures, namely a thin 2D molybdenum disulfide (MoS_2_) twisted flake and a 1D MoO_3_/MoS_2_ nanorod heterostructure. In both cases, we can successfully identify the contributions to the diffraction pattern from individual crystal structures and materials using the EWPC and evaluate the associated strain fields corresponding to well‐separated structures, layers, or materials.

Although our focus is on vdW materials, specifically the TMD MoS_2_, our method is highly versatile and can be applied to strain‐field characterization in other low‐dimensional nanostructured materials. Furthermore, while we developed our method for 4D‐STEM datasets captured with the EMPAD, it generally applies to any 4D‐STEM dataset captured by one of the many types of 4D‐STEM detectors currently available. Our approach offers a new avenue for investigating the intricate relationship between non‐trivial nanoscale strain patterns and the resulting optical, electronic, magnetic, and mechanical properties of nanostructured materials.

## 2 Results and Discussion

### 2.1 Nanobeam Electron Diffraction (NBED) and the Exit‐Wave Power Cepstrum (EWPC)

Obtaining accurate lattice parameters is crucial for strain measurements in thin nanomaterials such as the vdW materials considered here. One way to determine the lattice parameters is by analyzing the diffraction pattern generated by the electron beam as it passes through the specimen. Classical diffraction techniques, like selected area diffraction (SAD), sample a large crystalline area, which averages the diffraction pattern and reduces precision.

A way forward is provided by converging the electron beam into a nanometer‐sized probe, a technique known as convergent beam electron diffraction (CBED). To determine lattice parameters using CBED, one can analyze the disk spacings in the zero‐order Laue zone (ZOLZ) pattern^[^ ^18^ ^]^ or measure the radius of the higher‐order Laue zone (HOLZ) rings.^[^ ^37^, ^38^ ^]^ However, the latter is usually only possible for thicker specimens or specific tilt angles.^[^ ^39^, ^40^ ^]^ Therefore, one commonly uses the disk spacing in the ZOLZ pattern to determine the lattice parameters. However, it is only visible under specific electron beam conditions known as nano‐beam electron diffraction (NBED).

Within an NBED pattern, the radius of the diffraction disks is smaller than the distance between the center points, which means that the convergent angle (*α*) of the electron beam is smaller than the Bragg angle (*θ*_B_) for the lattice planes, causing the disks to separate. To achieve NBED conditions, a small condenser aperture (between 10 and 50 μm) can be used.^[^ ^41^ ^]^ Thus, by combining NBED conditions in STEM imaging with a pixelated detector, the ZOLZ pattern can be recorded for each location on the specimen, allowing for accurate determination of the lattice parameters with excellent spatial resolution.

**Figure 1:**
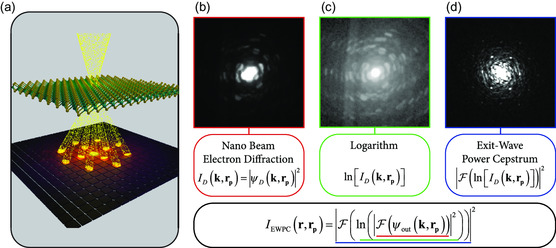
Determination of the exit‐wave power cepstrum. a) Schematic representation of the STEM imaging system, including the pixelated detector, where the electron beam probes a 2D material specimen, and the diffracted beams form an NBED pattern onto the pixelated detector. b) A full nanobeam electron diffraction pattern of internally twisted WS_2_, which provides the starting point for generating the EWPC. c) The logarithm of the NBED pattern displayed in c, the second step in generating the EWPC. d) The resulting EWPC is generated by taking the fast Fourier transform of c.

**Figure** **1**a displays a schematic representation of the STEM imaging system, complete with a pixelated detector. The system operates by directing an electron probe toward a thin 2D material specimen, leading to the formation of diffracted beams that form an NBED pattern on the pixelated detector. In such a configuration, the electron beam probes the specimen at position **r**_p_ = (*x*,*y*). The projection lenses, not depicted in Figure 1a, transmit the corresponding exiting electron wavefunction, $\psi_{out}\left(k,r_{p}\right)$, onto the pixelated detector. These lenses perform an effective Fourier transformation on the electron wave such that the electron wave at the detector plane can be expressed^[^ ^42^ ^]^ as

(1) $$\psi_{D}\left(k,r_{p}\right)=ℱ\left\{\psi_{out}\left(r,r_{p}\right)\right\}\left(k\right)\;,\;\;k=(k_{x},k_{y})$$

The electron wave intensity as measured at the detector plane is then given by $I_{D}\left(k,r_{p}\right)={\left|\psi_{D}\left(k,r_{p}\right)\right|}^{2}$. Figure 1b shows an example of a captured electron wave intensity pattern, which corresponds to an NBED pattern in this case. This NBED pattern is taken from a internally twisted WS_2_ bulk crystal.^[^ ^43^ ^]^ The intensity distribution in such an NBED pattern directly relates to the crystal lattice parameters. However, this pattern is also susceptible to variations in the specimen thickness and to the tilt or orientation of the specimen, as can be seen in Figure 1c.

Indexing an NBED pattern works best when the specimen is precisely oriented in zone‐axis (ZA).^[^ ^44^ ^]^ However, since the goal is to measure local variations in the lattice parameters of micrometer‐sized specimens with many grain boundaries, it is impossible to align the entire specimen in ZA. To overcome this challenge, we apply the EWPC approach, developed by Padget et al.,^[^ ^31^, ^45^ ^]^ which enables us to isolate the intensity contributions of the crystal lattice in the NBED pattern and filter out any contributions from non‐ideal sample orientation. The EWPC pattern contains sharp peaks representing the inter‐atomic spacing of the crystal, which is ideal for measuring small variations in the crystal lattice parameters of the specimen.

An EWPC can be generated from an NBED pattern using the following relation

(2) $$I_{\text{EWPC}}\left(r,r_{p}\right)={\left|ℱ\left(\ln{\left|ℱ\left(\psi_{\text{out}}\left(k,r_{p}\right)\right)\right|}^{2}\right)\right|}^{2}$$

the initial step in Equation (2) involves taking the Fourier transform of the electron wavefunction as it exits the specimen. The microscope lenses automatically perform this transformation, resulting in the NBED pattern depicted in Figure 1b. This Fourier transformation translates any convoluted signals into a multiplication of signals.

Next, we construct the EWPC from the NBED pattern in Figure 1b by taking the logarithm of the intensity, as shown in Figure 1c. Through the application of a logarithmic transformation, the intensity range within the NBED image is effectively flattened, revealing additional Bragg reflections. Furthermore, the logarithm isolates the contributions of the convoluted signals within the wavefunction (additional details can be found in Section S1, Supporting Information).

Finally, a fast Fourier transform (FFT) is employed to convert the logarithmic NBED pattern into a real‐space representation of the inter‐atomic distance. The EWPC pattern in Figure 1d reveals six peaks arranged in a hexagonal pattern, characteristic of transition metal dichalcogenide (TMD) materials. Even though the NBED pattern in Figure 1c was not oriented along the ZA or any high‐symmetry direction, the hexagonal atomic arrangement can still be clearly identified in the EWPC. The short‐range contributions of the non‐ideal sample tilt center around the middle of the EWPC, and the sharp peaks in the EWPC represent the contributions of the TMD crystal lattice. By applying band‐pass filtering, one can display the individual contributions of the tilt and the crystal lattice in the NBED. See Section S2, Supporting Information, for more details.

### 2.2 Analyzed 2D and 1D MoS_2_ Nanostructures

While our method is applicable to and can be utilized for the strain‐field characterization of a broad range of crystalline materials,^[^ ^46^, ^47^, ^48^ ^]^ here we showcase its potential for the specific case of the TMD materials MoS_2_. In particular, we investigate two unique morphologies of the vdW material MoS_2_: a 2D MoS_2_ thin film and a 1D MoS_3_/MoS_2_ nanorod heterostructure. All these morphologies were synthesized directly on a Si_3_N_4_ TEM grid using chemical vapor deposition (CVD).^[^ ^49^, ^50^, ^51^ ^]^ For more information about the synthesis of the MoS_2_ specimens, please refer to Section S3, Supporting Information.

The first nanostructure consists of single‐layer MoS_2_ with a layer thickness of 0.65 nm,^[^ ^52^, ^53^ ^]^ significantly thinner than the 5 nm thick Si_3_N_4_ membrane that spans the TEM grid. This thin MoS_2_ layer produces a sizable NBED pattern suitable for strain measurements. However, in the thinnest regions of the MoS_2_ specimen, the NBED diffraction pattern is barely distinguishable from the background signal of the amorphous Si_3_N_4_ substrate. Section S11, Supporting Information, illustrates the variation in NBED pattern intensity between the thinnest and thickest parts of the MoS_2_ specimen. Due to this limited diffraction contrast, conventional strain mapping techniques, which rely on detecting slight perturbations in the NBED diffraction pattern using disk detection, face challenges in accurately determining diffraction disk positions. Fortunately, even in the thinnest regions of the MoS_2_ specimen, the EWPC method generates sufficient contrast to accurately measure small perturbations. This quality makes the MoS_2_ specimen an ideal candidate for demonstrating the efficacy of the approach presented in this work, despite the initial limitations.

The second nanostructure examined is a MoO_3_ nanorod covered with a MoS_2_ shell, once again directly grown on a Si_3_N_4_ TEM grid using CVD.^[^ ^54^, ^55^ ^]^ The dimensions of the nanorods present a challenge in aligning the entire specimen along theZA, which is typically required for strain field measurements based on NBED. Utilizing the EWPC, our method eliminates this requirement, making it particularly suitable for measuring strain in the MoO_3_ nanorod. Additionally, horizontal MoS_2_ films can be found in the vicinity of the MoO_3_ nanorod on the substrate. This presents an opportunity to showcase the effectiveness of our method in selectively measuring strain in multiple nanostructures simultaneously.

### 2.3 Peak Tracking and Clustering

A single EWPC provides information on the inter‐atomic spacing of the crystal lattice directly beneath the electron probe. By scanning the electron probe across the entire specimen in a 2D grid, a 2D EWPC can be generated for each probe position **r**_p_. The resulting 4D dataset contains all the information necessary to determine the inter‐atomic spacing for each probe position on the entire specimen.

Within our approach, one evaluates the local strain variations by comparing the measured atomic spacing across the specimen with respect to a reference area. These strain fields are calculated through a four‐step process. First, we identify the approximate position of the sharp peaks for each EWPC in the acquired dataset. Next, we superimpose all the peak positions from every EWPC to form a weighted point cloud. We cluster the points in this point cloud into groups that match the inter‐atomic spacing of the crystal lattice. Subsequently, we determine the exact sub‐pixel location of the peaks within two non‐parallel clusters by calculating the center‐of‐mass (CoM) of the intensity in the EWPC. Finally, we evaluate the strain across the specimen by measuring the shift in the crystal spacing, represented by the sub‐pixel location of the EWPC peaks, with respect to the crystal spacing in the reference region.

**Figure 2:**
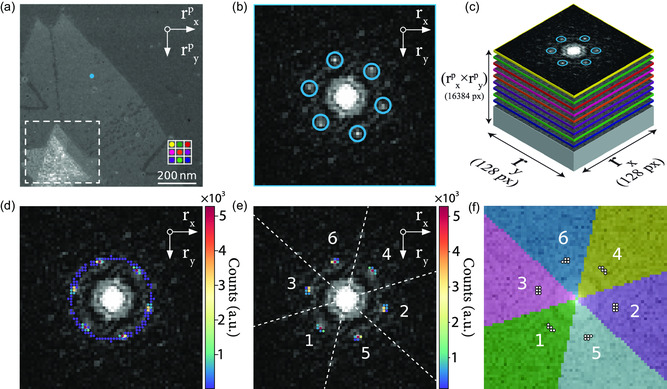
Tracking and clustering of EWPC peaks. a) ADF image of the MoS_2_ thin film studied in this work. The center of the nanostructure, contained in the white box, is composed of multiple overlapping, twisted MoS_2_ layers. Each pixel in this ADF image represents a different electron probe position. b) The EWPC corresponding to the blue dot in a, displaying the characteristic hexagonal atomic arrangement of MoS_2_. c) Representation of the stacking of EWPC patterns into a 2D data array with the *r*_*x*_ and *r*_*y*_ coordinates of every EWPC peak in the 4D dataset. d) A weighted point cloud, where each spot represents a unique peak location in the stacked dataset. The number of times a unique peak location occurs is indicated by the spot's color. e) A filtered point cloud, where only the peak locations that appear 53 times or more are displayed. The remaining points can be clustered into six groups. f) The resulting six clusters are generated by the *K*‐means clustering algorithm, where each identified peak position is assigned to one of the clusters.

**Figure** **2** illustrates the process of identifying the peak locations and the subsequent clustering of the point cloud. The reconstructed annular dark‐field (ADF) image in Figure 2a showcases the MoS_2_ thin film over an Si_3_N_4_ substrate, as described earlier. Each pixel in this ADF image represents the integrated intensity of a single NBED pattern at a distinct probe position while using an annular mask (see Section S4, Supporting Information, for additional details). An EWPC can be generated for each probe position using Equation (2). The EWPC for the probe location marked by the blue dot in Figure 2a is displayed in Figure 2b. This EWPC exhibits the characteristic hexagonal atomic arrangement of MoS_2_. To assess variations in the atomic arrangement (crystal lattice constants), it is necessary to automatically determine the location of these six peaks for every probe position.

Due to noise in the original NBED pattern, which is transferred to the EWPC, accurately detecting the peaks by identifying local maxima in Figure 2b is not feasible. Instead, we apply a difference of Gaussian (DoG) detection algorithm that blurs the EWPC with increasing standard deviations.^[^ ^56^ ^]^ This approach allows the peaks to be detected by identifying the local maxima in the difference between two successively blurred EWPCs (see Section S5, Supporting Information, for more details). Masking can be implemented to ensure that the detected peaks all fall within a specified annular region. Utilizing the annular mask ensures that the short‐range contributions of the sample tilt are entirely disregarded during the detection scheme. The DoG detection scheme is then repeated for each EWPC corresponding to every recorded probe position. By utilizing Dask arrays instead of the more commonly used Numpy arrays, the detection scheme can be executed for multiple EWPCs in parallel, significantly accelerating the entire process.

Upon detecting the peaks in every EWPC within the 4D dataset, an array is obtained that lists the *r*_*x*_ and *r*_*y*_ coordinates of the EWPC peaks for each electron probe position. Subsequently, we combine the arrays for each probe position to form a single 2D array listing all the *r*_*x*_ and *r*_*y*_ coordinates for every EWPC peak throughout the entire 4D dataset, as illustrated schematically in Figure 2c. We identify the unique peak positions in the list and determine the number of occurrences to create a weighted point cloud, as displayed in Figure 2d. The spots within the point cloud correspond to a unique peak location, with the color of each spot indicating how often this peak location is detected. The occurrence of hotspots within the point cloud is expected, given that strain effects represent a perturbation rather than a drastic shift in the atomic arrangement of the MoS_2_ nanostructure. The remaining spots in the point cloud only appear a few times and can be attributed to incorrectly tracked peaks originating from local maxima in the EWPC noise.

Figure 2e shows a point cloud with every spot that appears less than 53 times filtered out, revealing a six‐fold symmetry that corresponds to the hexagonal atomic arrangement observed in the single EWPC from Figure 2b. By applying a *K*‐means clustering algorithm to the filtered point cloud, the spots can be grouped into six clusters, with each peak position assigned to one of the clusters, as shown in Figure 2f. All peaks with coordinates in the same cluster are assumed to be a shifted or rotated version of the same peak.

### 2.4 Determination of Strain Field Maps

The DoG peak identification algorithm determines the peak positions with single‐pixel accuracy. However, to achieve the best accuracy in the strain map, it is necessary to determine the center of the peaks with sub‐pixel accuracy. To do this, we use the peak position found by the DoG scheme as the center of a small circular mask, with a radius *R*_m_ of two pixels, that surrounds the peak. Section S6, Supporting Information, motivates the need for sub‐pixel accuracy and the choice of *R*_m_ = 2 to achieve the most accurate strain maps. Subsequently, the sub‐pixel maximum can be determined by calculating the CoM of the EWPC intensity within this circular mask. To calculate the local strain variations in the specimen, it is sufficient to track the changes in the peak position of two peaks that are not parallel. Thus, calculating the sub‐pixel position requires only the peaks from two non‐parallel clusters as input, which reduces the computation time.

**Figure 3:**
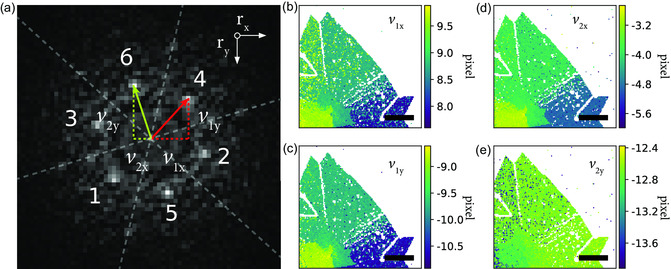
EWPC peak position maps with sub‐pixel accuracy. a) The same EWPC as in Figure 2b, now with the vectors **v**_1_ and **v**_2_ indicating the positions of the peaks in clusters 4 and 6 for this specific EWPC. b–e) Maps displaying the *x* and *y* coordinates, in pixels, for the same **v**_1_ and **v**_2_ vectors for each probe position. The coordinate origin, O(0,0), is the center of the EWPC, and the scale bars in (b–e) are 200 nm.

**Figure** **3** illustrates how the *r*_*x*_ and *r*_*y*_ coordinates of two peaks in the EWPC can be mapped for every probe position. Firstly, we select only the peaks corresponding to clusters 4 and 6 from Figure 2f. Subsequently, we determine the *r*_*x*_ and *r*_*x*_ coordinates of the two non‐parallel peaks by calculating their sub‐pixel maxima. The resulting vectors, denoted by **v**_1_ and **v**_2_, represent the location of the two EWPC peaks. The *r*_*x*_ and *r*_*y*_ coordinates of the vectors are given in pixels, and the center of the EWPC image serves as the origin, as depicted in Figure 3a. The maps in Figure 3b–e depict the *r*_*x*_ and *r*_*y*_ coordinates for **v**_1_ and **v**_2_ for every probe position.

Using the information contained in these two vectors, one can calculate the relative strain with respect to the reference area of the specimen for each probe position. To calculate the strain fields, we adopt the method commonly used in GPA for finite displacements^[^ ^57^ ^]^ and construct a deformation matrix **D**. Depending on the preferred coordinate basis, the deformation matrix can be calculated using

(3) $$D_{car}=AA_{0}^{-1}\;\;\text{or}\;D_{vec}=A_{0}^{-1}A$$

Here, matrix **A** comprises the measured vectors ${v^{\prime }}_{1}$ and ${v^{\prime }}_{2}$, while matrix 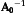 contains the reference vectors **v**_1_ and **v**_2_. The subscripts “**car**” and “**vec**” in **D**_**car**_ and **D**_**vec**_ indicate which basis determines the deformation. **D**_**car**_ describes the deformation using the Cartesian coordinates system as a basis. In contrast, **D**_**vec**_ characterizes the deformation with respect to the basis formed by vectors **v**_1_ and **v**_2_. Consequently, **D**_**vec**_ describes the deformation perpendicular to the lattice planes represented by the selected EWPC peaks. The choice of basis depends on the specific requirements of the analysis. In this work, we solely demonstrate the deformation concerning the vector basis (**D**_**vec**_). The 2 × 2 matrix **D**_**vec**_ entirely describes the affine transformation between the vectors ${v^{\prime }}_{1}$ and ${v^{\prime }}_{2}$ in the measured region and the vectors **v**_1_ and **v**_2_ in the reference region of the specimen as shown below

(4) $$\left[\begin{array}{cc} {v^{\prime }}_{1x} & {v^{\prime }}_{2x}\\ {v^{\prime }}_{1y} & {v^{\prime }}_{2y} \end{array}\right]=D_{vec}\cdot \left[\begin{array}{cc} v_{1x} & v_{2x}\\ v_{1y} & v_{2y} \end{array}\right]\;.$$

The deformation matrix **D**_**vec**_ contains information about the angular rigid rotation of the specimen and strain deformations within the specimen. By performing a polar decomposition, one can separate the rotation from the strain components in the matrix **D**_**vec**_. Refer to Section S1, Supporting Information, for further details on the definition of **D**_**car**_ and **D**_**vec**_, the different coordinate bases, and the polar decomposition.

**Figure 4:**
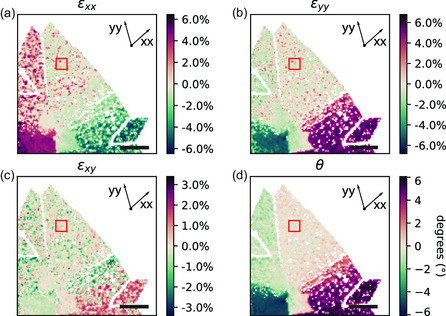
Strain and rotation maps of a MoS_2_ thin film nanostructure. These maps correspond to the nanostructure analyzed in Figures 2 and 3, with the reference area marked by a red box in each panel. a) Strain along the *xx*‐direction, featuring tensile strain $\left(\varepsilon_{xx}≃3\%\right)$ on the left side of the MoS_2_ nanostructure and compressive strain $\left(\varepsilon_{xx}≃-3\%\right)$ on the right side. b) Strain along the *yy*‐direction, displaying compressive strain $\left(\varepsilon_{yy}≃-3\%\right)$ on the left side of the MoS_2_ nanostructure and tensile strain $\left(\varepsilon_{yy}≃4\%\right)$ on the right side. Note that the tensile strain is consistently perpendicular to the direction of the crack in the MoS_2_ nanostructure. c) Minimal shear strain $\left(\varepsilon_{xy}\leq 1\%\right)$. d) The rigid rotation map illustrates a clockwise rotation of the entire MoS_2_ nanostructure. Scale bars in all figures represent 200 nm.

By calculating the deformation matrix and executing a polar decomposition for each probe position, we generate the strain and rotation maps displayed in **Figure** **4**. Specifically, Figure 4a,b provide the strain components, with *ε*_*xx*_ representing the strain in the *xx* direction, which is oriented along the direction of cluster 4 $\left\langle 10\bar{1}0\right\rangle$ in Figure 3a, and *ε*_*yy*_ representing the strain in the *yy* direction, which is oriented along the direction of cluster 6 $\left\langle 11\bar{2}0\right\rangle$ in Figure 3a. Figure 4c corresponds to the shear strain (*ε*_*xy*_), and Figure 4d displays the map of rigid rotation *θ* within the specimen. All strain and rotation maps have a small Gaussian filter applied to smooth out the pixelated nature of our method. This leads to a more realistic strain field by eliminating point outliers. Further details on the Gaussian filter's effects can be found in Section S6, Supporting Information, while unfiltered strain maps are available in Section S8, Supporting Information. The red box in each panel indicates the reference region employed for the strain calculations.

The rotation map in Figure 4d reveals a rigid clockwise rotation of the MoS_2_ nanostructure relative to the reference region. In contrast, Figure 4a,b (representing the *ε*_*xx*_ and *ε*_*yy*_ components) indicate substantial strain in the *xx* and *yy* directions, respectively. In particular, relative to the reference area, the nanostructure exhibits tensile strain in the *xx* direction on the left side $\left(\varepsilon_{xx}≃3\%\right)$ and compressive strain on the right side $\left(\varepsilon_{xx}≃-4\%\right)$. Conversely, for the strain in the *yy* direction and in relation to the same reference area, we observe compressive strain on the left side $\left(\varepsilon_{yy}≃-3\%\right)$ and tensile strain on the right $\left(\varepsilon_{yy}≃4\%\right)$. Additionally, on both the left and right sides of the nanostructure, the tensile strain is observed to be perpendicular to the crack present within the nanostructure. Consistently, the magnitude of strain we observed perpendicular to the direction of the crack in the MoS_2_ layer and the rotation across the entire nanostructure align with previously reported behaviors in MoS_2_ monolayers, which have been attributed to the emergence of crack structures due to point defects.^[^ ^58^, ^59^ ^]^ Furthermore, in the *xx* direction, distinct regions displaying tensile strain, approximately $\left(\varepsilon_{xx}≃2\%\right)$, are evident. These strained regions can be predominantly linked to the vicinity of point defects and holes present in the MoS_2_ layer, a visualization of which is provided in Figure 4a. We have estimated the accuracy of the strain and rigid rotation maps to be around ±1.1% and ±4.47°, respectively, as detailed in Section S6, Supporting information. It is worth noting that the precision in strain map readings might be further enhanced by increasing the spatial separation between the EWPC peaks and the central position of the EWPC pattern. The strain maps in Figure 4 demonstrate that our method is able to accurately determine the strain in thin TMD materials with weak diffraction contrast. Our method achieves this without the need to filter out the strong contributions of the amorphous Si_3_N_4_ background, which can influence the accuracy of other strain mapping methods, as discussed in Section S11, Supporting Information.

### 2.5 EWPC‐Based Strain Measurements in Twisted MoS_2_ Flakes and 1D MoS_2_/MoO_3_ Nanorod Heterostructures

The preceding discussion highlights the capabilities of our approach, which relies on tracking all EWPC peaks using a difference of Gaussians (DoG) scheme, clustering the identified peaks, and calculating the sub‐pixel maxima for two non‐parallel clusters in determining local strain fields in MoS_2_ monolayers. A key aspect of this approach, which makes it applicable to a broad class of nanomaterials and geometries, lies in the clustering of the EWPC peaks. Indeed, by selecting two different clusters, the strain calculation can be performed for two distinct peaks in the EWPC without having to track the complete set of EWPC peaks again, significantly reducing processing time. Furthermore, this flexibility also renders our method suitable for measuring strain fields in nanostructures with geometries different from the thin film configuration discussed earlier. For instance, in multilayer materials with stacked twisted layers, Moiré patterns can form. These Moiré patterns represent the atomic arrangement of two atomic layers of the same material that are slightly rotated with respect to each other. In such a configuration, the atomic arrangements of the different twisted layers appear as separate peaks in the EWPC.

To demonstrate the applicability of our method in identifying Moiré patterns in vdW materials, we observe that the MoS_2_ nanostructure examined in Figure 2, 3, 4 also includes a small region comprising multiple stacked atomic layers. This region is located at the center of the nanostructure and is highlighted by the white box in Figure 2a. Additionally, we extend our study to twisted heterostructures MoSe_2_/WSe_2_, the results of which can be found in Section S7, Supporting Information. In the following, we primarily focus on Moire patterns arising from the same material.

**Figure 5:**
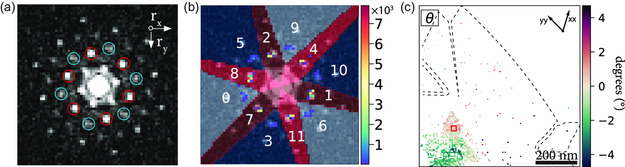
Moiré pattern analysis of twisted multilayer MoS_2_ films. a) EWPC for the same MoS_2_ specimen studied in Figure 2, 3, 4, corresponding to the area displaying a twisted multilayer structure and indicated by the white box in Figure 2a. The resulting Moiré pattern consists of two sets of six peaks, each set belonging to an individual atomic layer. b) The resulting weighted point cloud obtained from the DoG peak detection algorithm, where all unique peak positions with at least 35 occurrences are included. This point cloud is divided into 12 clusters, with the red clusters belonging to the first layer and the blue clusters belonging to the second layer. c) The rotation map of the second layer, generated by selecting only the peaks in clusters 9 and 5, with the reference area indicated by the red box.

**Figure** **5**a presents an EWPC obtained from a multilayer region containing two sets of six peaks, with each set belonging to a separate stacked layer. By tracking the 12 peaks simultaneously, a single point cloud can be generated (Figure 5b), representing all the peaks associated with both layers. Two peaks from the same layer can then be chosen to isolate the strain fields and the rigid rotation angle associated with each layer. For instance, selecting clusters 4 and 2 in Figure 5b isolates the same MoS_2_ layer as in Figure 4, yielding a strain map that, as expected, is comparable to the previous results (see Section S8, Supporting Information). Conversely, selecting clusters 9 and 5 isolates the second MoS_2_ layer, which is only present in the region of the specimen highlighted by the white box in Figure 2a. Figure 5c displays the resulting rotation map for this second layer, demonstrating how our method can also be used to independently measure the rigid rotation deformation arising in the crystal lattice of each layer in the stacked vdW multilayer. The corresponding strain maps of the second layer can be found in Section S8, Supporting Information.

**Figure 6:**
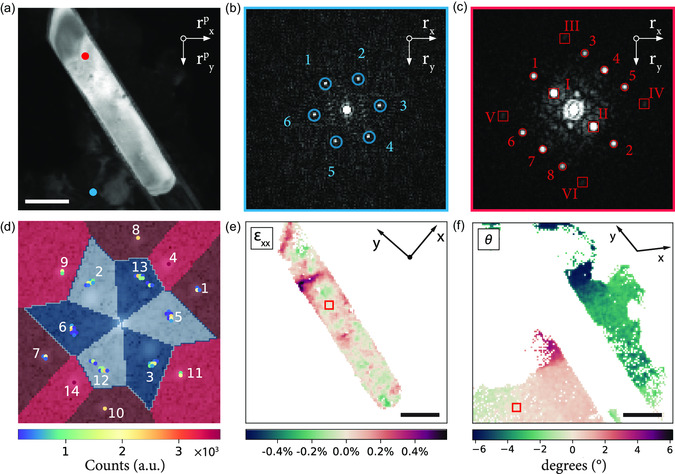
Advanced clustering analysis with DoG for non‐planar 2D materials. a) ADF image of a 1D MoO_3_/MoS_2_ nanorod with horizontal growth of MoS_2_ observed on the substrate. The low contrast in the image makes it difficult to discern the details of the structure. b) The EWPC obtained from a probe position on the MoS_2_ film, indicated by the blue dot in (a). c) The EWPC obtained from a probe position on the MoO_3_ nanorod, marked by a red dot in (a). d) The weighted point cloud of all unique peak positions (occurring at least 150 times) identified by the DoG detection algorithm, which can be classified into 14 clusters. The blue clusters correspond to peaks from the MoS_2_ film and exhibit the same six‐fold symmetry as in (b), while the red clusters correspond to the peaks from the MoO_3_ nanorod and display mirror symmetry as in (c). e) Shear Strain map, *ε*_*xy*_, calculated from clusters 4 and 9 and representing the region within the MoO_3_ nanorod. f) The rotation map *θ*, of the MoS_2_ thin film was determined by using the information in clusters 13 and 2. The scale bars in (a,e,f) correspond to 50 nm.

Our method can also be applied to heterostructures composed of different materials with non‐trivial geometrical configurations, as demonstrated in the following example. **Figure** **6**a displays an ADF image of a MoO_3_ nanorod surrounded by a thin MoS_2_ shell, resulting in a 1D MoO_2_/MoO_3_ heterostructure. In the vicinity of the nanorod on the substrate, we also observe the horizontal growth of MoS_2_. In the ADF image, the contrast is quite low, making it challenging to discern the details of the structure.

Figure 6b depicts the EWPC corresponding to the region displaying horizontal MoS_2_ growth, located at the bottom left corner and marked by a blue dot in Figure 6a. The EWPC highlights the characteristic hexagonal peak distribution associated with horizontally‐oriented MoS_2_. Figure 6c showcases the EWPC captured at a point in the center of the nanorod, revealing a total of 14 peaks. Four of these peaks, labeled with Roman numerals (III–VI), exhibit low intensity, making their detection challenging. Thus, we exclude them before implementing the automated DoG detection scheme. Additionally, the peaks labeled as I and II are located in close proximity to the center of the EWPC and to the peaks corresponding to the horizontal MoS_2_ film. To prevent them from clustering together with the peaks related to the horizontal growth during the DoG detection, we exclude these peaks as well. As a result, we limit the maximum number of peaks the DoG detection scheme needs to identify to eight peaks. This approach enables us to detect all peaks associated with the region of horizontal MoS_2_ film (indicated by a blue circle in Figure 6b) and the peaks associated with the MoO_3_ nanorod (indicated by a ref circle in Figure 6c).

Figure 6d presents the resulting point cloud, showcasing all peaks detected more than 150 times after applying the DoG detection scheme. We can identify 14 clusters, where the blue clusters represent the hexagonal pattern of the horizontal MoS_2_ film, and the red clusters correspond to the MoO_3_ nanorod. The capability to cluster both strain and rigid rotation information offers a significant advantage, as it enables the isolation of the individual contributions of the MoO_3_ nanorod and the MoS_2_ horizontal film. For example, by selecting two clusters that are part of the same pattern, our method can effectively distinguish between the strain fields present in the MoO_3_ nanorod and those associated with the MoS_2_ horizontal film. Figure 6e,f provide an illustration of this concept. Figure 6e showcases the shear strain map (*ε*_*xy*_) of the MoO_3_ nanorod, calculated by selecting clusters 4 and 9. The map highlights that the nanorod has a region of tensile strain (*ε*_*xx*_ = 0.52) at the top of the nanorod. This observation is consistent with the change in intensity in the ADF image in Figure 6a, which indicates a sharp change in the thickness of the MoO_3_ nanorod.

In contrast, Figure 6f illustrates the rigid rotation angle (*θ*) in the MoS_2_ thin film, obtained by selecting clusters 13 and 2. The map unveils the presence of two separate MoS_2_ films in proximity to the nanorod. Notably, the MoS_2_ film located to the right of the nanorod manifests a pronounced rigid rotation compared to the film on its left. This differential rotation suggests that the MoS_2_ films were grown independently and are not intrinsically linked to the MoO_3_ structure. Building on these findings, our results showcase the ability of our method to independently measure strain fields across diverse materials encapsulated within intricate heterostructures and geometries. This ability is currently beyond the scope of other 4D‐STEM strain mapping techniques.^[^ ^23^, ^31^ ^]^ For a comprehensive examination of the dataset, inclusive of strain and rigid rotation maps pertinent to both the MoO_3_ nanorod and the MoS_2_ film, see Section S9, Supporting Information.

## 3 Summary and Outlook

In this work, we present a novel approach based on 4D STEM imaging with a pixelated detector (EMPAD) for determining local strain fields in 2D vdW materials with nanoscale resolution over complete micron‐sized specimens. This method accurately characterizes strain fields and rotation angles, complementing and extending the capabilities of existing strain measurement techniques. A notable advantage is its capability to differentiated between the strain contributions of adjacent layers or distinct materials within a heterostructure. As such, it emerges as an indispensable tool for automating the detection of Moiré patterns, in addition to measuring strain fields and rigid rotations in layered vdW nanomaterials.

While our investigation focus centered on MoS_2_ in varied configurations, the strategy is fully general and suitable for strain‐field characterization in other complex nanomaterials beyond the 2D vdW family. The application of this approach can revolutionize our understanding of the nexus between nanoscale strain and the consequent electronic and magnetic attributes of a wide range of nanomaterials. Especially when coupled with spatially‐resolved measurements like the band gap and the dielectric function procured from electron energy loss spectroscopy (EELS) on the same specimen, as demonstrated for the case of internally twisted WS_2_ nanostructures.^[^ ^43^ ^]^ The corresponding software framework, dubbed StrainMAPPER, is now available to the global community via its GitLab repository, complemented with comprehensive documentation and illustrative examples. We anticipate that this method will become a valuable resource for scientists aiming to understand the implications of nanoscale strain fields on the properties of the materials under study, a key step in bridging the gap between fundamental vdW material science and their implementation in technological applications.

## 4 Experimental Section

4.1

4.1.1

##### Transmission Electron Microscope Experiments

All 4D‐STEM measurements were conducted using a Titan Cube microscope operated at 300 kV, combined with an EMPAD optimized for 4D‐STEM. The dwell time for all measurements was set at 1 ms. The resolution of all the 4D‐STEM images was 128 × 128, resulting in a 128 × 128 × 128 × 128 dataset. A 10 μm C2 aperture was used for all measurements, yielding a convergence angle of 0.53 mrad. The camera length used for the measurements on the MoS_2_ nanostructure from Figure 2a was 460 mm, while the camera length used for the measurements on the MoO_3_ nanorod from Figure 6a was 285 mm. See Section S10, Supporting information for an overview.

##### Peak Tracking and Clustering Algorithms

The proposed approach involves multiple steps, including EWPC peak tracking with a DoG detection scheme, *K*‐means clustering of unique peak positions, calculating the sub‐pixel maximum using the CoM, and affine transformation determination relative to a reference area. The method is built on the Pixstem Python package,^[^ ^60^ ^]^ which loads and processes 4D datasets into memory‐optimized Dask arrays.^[^ ^61^ ^]^ Using Dask arrays enables parallel CPU computing to significantly accelerate all operations in our approach.^[^ ^62^ ^]^ This makes it possible to efficiently use our method on a personal computer with just 8 GB of system memory and four multi‐threading CPU cores. The generation of the EWPCs from the NBED patterns, and basic operations on the EWPCs, is accomplished by combining custom code and using existing Python packages. The DoG detection scheme and the *K*‐means clustering are provided by the scikit image^[^ ^63^ ^]^ and learn^[^ ^64^ ^]^ Python packages, respectively. The sub‐pixel maximum is calculated using a CoM algorithm provided by Pixstem along with a modified mask generation algorithm optimized for 4D masks. We determine the final affine transformation and strain maps using our custom code, which can calculate the deformation with respect to the Cartesian or vector basis. The polar decomposition is performed using SciPy.^[^ ^65^ ^]^

Additionally, we have conducted a comparison analysis of our proposed approach with alternative strain mapping methods, including py4DSTEM^[^ ^23^ ^]^ and PC‐STEM.^[^ ^31^ ^]^ This comparison was performed using two distinct datasets, and the detailed results can be found in Section S11, Supporting Information.

## Conflict of Interest

The authors declare no conflict of interest.

## Code Availability

The code used to produce the results of this study, including detailed documentation and instructions for each process step, is available in the StrainMAPPER repository and can be accessed via this link https://gitlab.tudelft.nl/conesabojlab/strainmapper. The open‐source code is available under the GNU GPL v3 license. Our package functions contain all the necessary steps to go from a 4D NBED dataset to strain maps.

## Author Contributions

M.B. synthesized the analyzed samples. M.B. conducted experiments, collected data, and developed the code and user interface for the strain mapping. M.B. and S.E.v.H. conducted data analysis and interpretation with input from S.C.‐B. S.C.‐B. and M.B. were responsible for writing the initial draft of the manuscript, with input and revisions from all authors. S.C.‐B., M.B., and S.E.v.H. all contributed to reviewing and editing the manuscript. S.C.‐B. supervised all aspects of the project. All authors have read and approved the final version of the manuscript.

## Supporting information

Supplementary Material

## Data Availability

The data that support the findings of this study are available from the corresponding author upon reasonable request.
