# Supplementary material for: 4D‐STEM Nanoscale Strain Analysis in van der Waals Materials: Advancing beyond Planar Configurations
Source: Small Sci. 2024 Jan 12;4(3):2300249. doi: 10.1002/smsc.202300249 (PMC11935214; doi:10.1002/smsc.202300249)
Supplement: Supplementary file 1 — Supplementary Material [file SMSC-4-2300249-s001.pdf]

Supporting Information: 4D-STEM Nanoscale Strain Analysis in van der Waals Materials: Advancing Beyond Planar Configurations

[1]Kavli Institute of Nanoscience, Delft University of Technology, 2628 CJ, Delft, The Netherlands
*Corresponding author. Email: s.conesaboj@tudelft.nl

Contents

S1 Equations for the exit-wave power cepstrum (EWPC) and the deformation matrix **2**
S1.1 The EWPC 2
S1.2 The deformation matrix 2

S2 EWPC bandpass filtering **6**

S3 Synthesis of the MoS_2 and MoO_3 nanostructures **7**

S4 Virtual annular dark field image **8**

S5 Difference of Gaussian method **9**

S6 Study on the systematic errors in the StrainMAPPER method **10**

S7 Twisted Heterostructures: $MoSe_2$/WSe_2 Study **15**

S8 Strain and rotation maps of the MoS_2 Moiré multilayer **19**

S9 Strain and rotation maps of the MoS_2/MoO_3 heterostructure **20**

S10 4D-STEM acquisition details **22**

S11 Comparison between 4D-STEM strain mapping methods **23**

S1 Equations for the exit-wave power cepstrum (EWPC) and the deformation matrix

S1.1 The EWPC

The Exit-Wave Power Cepstrum (EWPC) can be derived from a nanobeam electron Diffraction (NBED) pattern using the following relation:

$$I_{\text{EWPC}}\left(\mathbf{r}, \mathbf{r}_p\right) = \left|\mathcal{F}\left(\ln\left|\mathcal{F}\left(\psi_{\text{out}}\left(\mathbf{k}, \mathbf{r}_p\right)\right)\right|^2\right)\right|^2. \tag{1}$$

The first Fourier transform of the exiting wavefunction ψ_{out} is automatically performed by the lens system in the microscope, forming the aforementioned NBED. This Fourier transformation converts any convoluted signals within the wavefunction into a multiplication of signals.

$$\mathcal{F}\left(u\left(t\right) * v\left(t\right)\right) = U\left(\omega\right) \cdot V\left(\omega\right) \tag{2}$$

The next step in generating the EWPC involves taking the logarithm of the wavefunction. This process isolates the contributions of the convoluted signals within the wavefunction, leading to a more refined analysis.

$$\ln\left(\left|U\left(\omega\right) \cdot V\left(\omega\right)\right|^2\right) = \ln\left(\left|U\left(\omega\right)\right|^2\right) + \ln\left(\left|V\left(\omega\right)\right|^2\right) \tag{3}$$

Lastly, the EWPC is obtained by performing the Fourier transform on Eq. 3

$$\text{EWPC} = F\left(\ln\left(\left|U\left(\omega\right)\right|^2\right) + \ln\left(\left|V\left(\omega\right)\right|^2\right)\right) = F\left(\ln\left(\left|U\left(\omega\right)\right|^2\right)\right) + F\left(\ln\left(\left|V\left(\omega\right)\right|^2\right)\right) \tag{4}$$

Eq. 4 shows how the EWPC can be used to separate convoluted contributions within the wavefunction, effectively isolating the effects of the crystal lattice while filtering out other influences. This selective isolation of crystal lattice contributions is particularly beneficial for accurate strain mapping.

S1.2 The deformation matrix

Let us define $\mathbf{v_1}$ and $\mathbf{v_2}$ as the basis vectors that span the object's initial reference configuration, and let $\mathbf{v}_1'$ and $\mathbf{v}_2'$ be the basis vectors that span the object's deformed configuration. The deformation matrix $\mathbf{D}$ describes the transformation that maps the reference matrix $\mathbf{A_0}$ onto the measured matrix $\mathbf{A}$. Where:

$$\mathbf{A_0} = \begin{bmatrix} v_{1x} & v_{2x} \\ v_{1y} & v_{2y} \end{bmatrix} \tag{5}$$

$$\mathbf{A} = \begin{bmatrix} v'_{1x} & v'_{1x} \\ v'_{1y} & v'_{1y} \end{bmatrix} \tag{6}$$

One can evaluate the deformation matrix **D** using the following two equations:

$$\mathbf{D_{car}} = \mathbf{A}\mathbf{A_0^{-1}} \rightarrow \mathbf{A} = \mathbf{D_{car}} \cdot \mathbf{A_0} \tag{7}$$

$$\mathbf{D_{vec}} = \mathbf{A_0^{-1}}\mathbf{A} \rightarrow \mathbf{A} = \mathbf{A_0} \cdot \mathbf{D_{vec}} \tag{8}$$

The subscripts "**car**" and "**vec**" in $\mathbf{D_{car}}$ and $\mathbf{D_{vec}}$ indicate the method used to calculate the deformation matrix. $\mathbf{D_{car}}$ involves inverting the reference matrix and multiplying it by the measured matrix, while $\mathbf{D_{vec}}$ involves multiplying the reference matrix by the inverted measured matrix. Both equations can be used to obtain a deformation matrix **D**, and both $\mathbf{D_{car}}$ and $\mathbf{D_{vec}}$ fully describe the affine transformation between the reference matrix $\mathbf{A_0}$ and the measured matrix **A**. However, $\mathbf{D_{car}}$ and $\mathbf{D_{vec}}$ describe the deformation in different reference frames.

This section discusses the difference between Eqs. 7 and 8. We begin by considering the definition of the strain matrix **E**:

$$\mathbf{E} = \begin{bmatrix} \varepsilon_{xx} & \varepsilon_{xy} \\ \varepsilon_{yx} & \varepsilon_{yy} \end{bmatrix}, \tag{9}$$

which is given by:

$$\mathbf{E} = \mathbf{U} - \mathbf{I} \text{ or } \mathbf{E} = \mathbf{V} - \mathbf{I}, \tag{10}$$

where the identity matrix represented as **I** is given by:

$$\mathbf{I} = \begin{bmatrix} 1 & 0 \\ 0 & 1 \end{bmatrix}$$

and **U** and **V** are the strain deformation matrices. These matrices represent the deformation of the specimen under strain and can be extracted from the general deformation matrix using a polar decomposition:

$$\mathbf{D} = \mathbf{R} \cdot \mathbf{U} = \mathbf{V} \cdot \mathbf{R} \tag{11}$$

where **R** is the ridged rotation matrix described by:

$$\mathbf{R} = \begin{bmatrix} \cos\theta & -\sin\theta \\ \sin\theta & \cos\theta \end{bmatrix}. \tag{12}$$

The polar decomposition indicates that any deformation of a specimen can be decomposed into a rigid-body rotation and a strain. Here, **U** and **V** represent the strain part of the deformation, while **R** represents the rigid-body rotation part of the deformation.

In order to highlight the differences between Eqs. 7 and 8, we assume a deformation with a vanishing rotation $\theta = 0$, resulting in $\mathbf{R} = \mathbf{I}$. Therefore Eq. 11 can be reduced to $\mathbf{D} = \mathbf{U} = \mathbf{V}$, which means that the strain matrix **E** can be expressed as:

$$\mathbf{E} = \mathbf{D} - \mathbf{I}, \tag{13}$$

which in turn can be rewritten as:

$$\mathbf{D} = \mathbf{E} + \mathbf{I}. \tag{14}$$

For example, let's assume a compressive strain of 25 % along the x-axis and a tensile strain of 50 % along the y-axis. In this case, the deformation matrix is given by:

$$\mathbf{D} = \begin{bmatrix} 0.75 & 0 \\ 0 & 1.50 \end{bmatrix} . \tag{15}$$

Note that the strain values of 25 and 50 % are not realistic, but they serve an illustrative purpose here. If now we apply this deformation matrix to Eq. 7 ($\mathbf{A} = \mathbf{D_{car}} \cdot \mathbf{A_0}$), we get:

$$\mathbf{A} = \begin{bmatrix} 0.75 & 0 \\ 0 & 1.50 \end{bmatrix} \cdot \begin{bmatrix} v_{1x} & v_{2x} \\ v_{1y} & v_{2y} \end{bmatrix} \cdot = \begin{bmatrix} 0.75 \cdot v_{1x} & 0.75 \cdot v_{2x} \\ 1.50 \cdot v_{1y} & 1.50 \cdot v_{2y} \end{bmatrix} \tag{16}$$

Eq. 16 shows that the vectors $\mathbf{v_1}$ and $\mathbf{v_2}$ are deformed along the Cartesian coordinates (x, y) by the deformation matrix $\mathbf{D_{car}}$. Every point x is compressed 0.75 times, while every point y is stretched 1.50 times. The green vectors in Fig. S1 schematically represent this deformation.

Now, if we apply the same deformation matrix to Eq. 8 ($\mathbf{A} = \mathbf{A_0} \cdot \mathbf{D_v}$), we get the result:

$$\mathbf{A} = \begin{bmatrix} v_{1x} & v_{2x} \\ v_{1y} & v_{2y} \end{bmatrix} \cdot \begin{bmatrix} 0.75 & 0 \\ 0 & 1.50 \end{bmatrix} = \begin{bmatrix} 0.75 \cdot v_{1x} & 1.50 \cdot v_{2x} \\ 0.75 \cdot v_{1y} & 1.50 \cdot v_{2y} \end{bmatrix} \tag{17}$$

As we can see from Eq. 17, the vectors $\mathbf{v_1'}$ and $\mathbf{v_2'}$ are now deformed along the direction of the original vectors. The new vector $\mathbf{v_1'}$ is compressed by 0.75 times along the direction of the original vector $\mathbf{v_1}$, while the new vector $\mathbf{v_2'}$ is stretched by 1.50 times along the direction of $\mathbf{v_2}$. This deformation effectively creates a new coordinate system (x', y') with vectors $\mathbf{v_1}$ and $\mathbf{v_2}$ as a basis. The red vectors in Fig. S1 schematically represent this deformation.

In summary, Eqs. 7 and 8 describe two different ways of representing the deformation of the specimen. Eq. 7 describes the deformation of the specimen with respect to the Cartesian coordinate system (x, y). The Cartesian basis is defined as the pixelated detector's x and y coordinates. Whereas, Eq. 8 describes the deformation with respect to the vector basis of $\mathbf{v_1}$ and $\mathbf{v_2}$. In our system, this basis is formed by the two EWPC peaks selected for the strain calculations. Using the vector basis, one can determine the strain along the two lattice planes represented by the EWPC peaks. The choice of which basis to use ultimately depends on the analysis's specific needs.

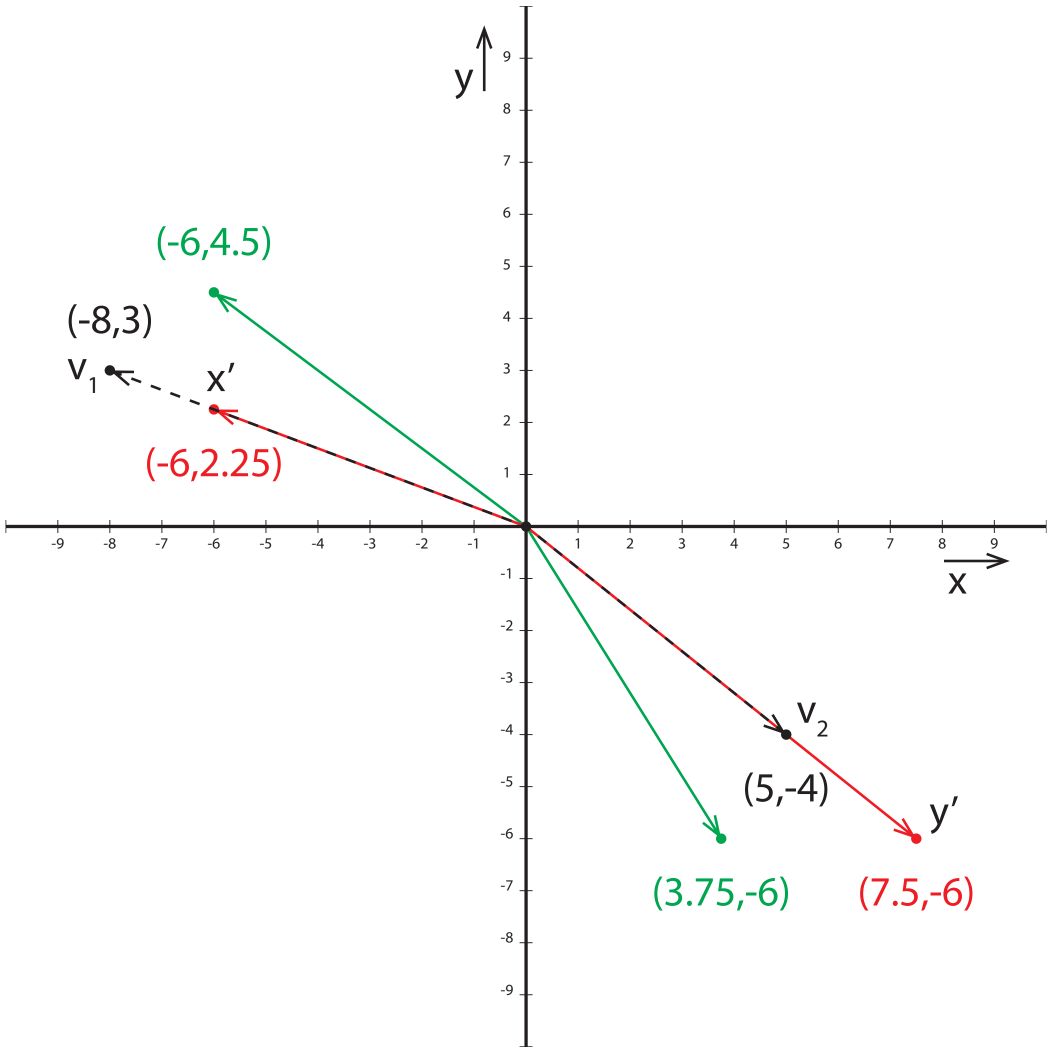

Figure S1: A schematic representation of a deformation applied along the Cartesian basis and along the vector basis. The two reference vectors $\mathbf{v_1}$ and $\mathbf{v_2}$ are denoted in black. A compressive strain of 25 % along the x-axis and a tensile strain of 50 % along the y-axis is applied. The green vectors represent the deformation applied in the Cartesian basis (x, y), while the red vectors represent the deformation applied in the vector basis (x', y').

S2 EWPC bandpass filtering

Fig. S2 provides further details on the EWPC Bandpass filtering.

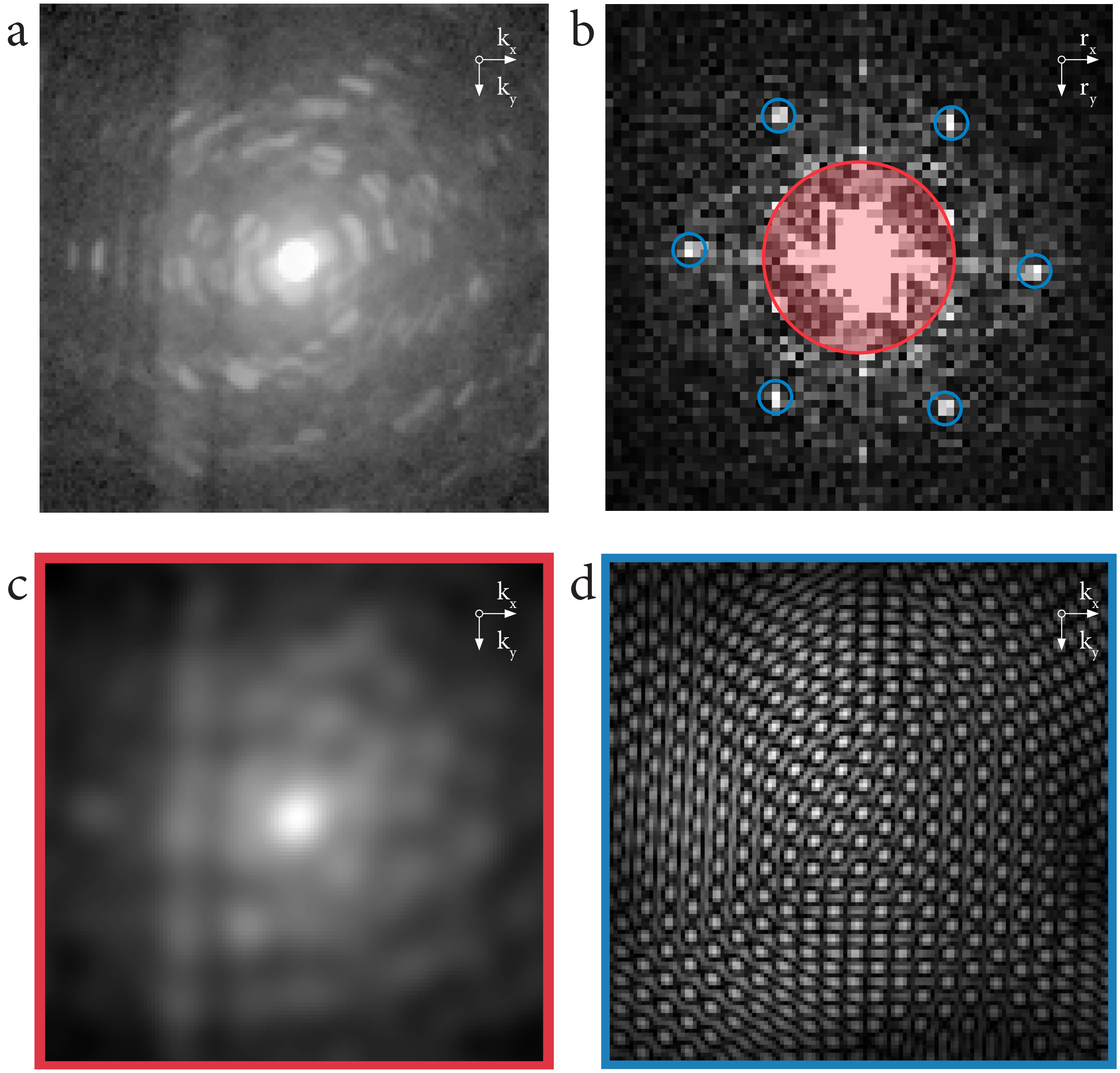

Figure S2: a) An NBED pattern of a typical TMD material. Similarly to Fig.1**c**, the intensity of the NBED pattern is normalized using a logarithm. **b)** The exit-wave power cepstrum (EWPC) of the NBED pattern in **a**. The short-range contributions to the intensity coming from the non-ideal sample tilt are located in the red circle. The sharp peaks, indicated by the blue circles, represent the contributions of the TMD crystal lattice. **c)** The inverse EWPC of the red area in **b**. This NBED contains contributions from the non-ideal two-dimensional sample, like sample thickness and tilt. **d)** The inverse EWPC of the blue areas in **b**. This NBED contains only the contributions of the TMD crystal lattice.

S3 Synthesis of the MoS_2 and MoO_3 nanostructures

The MoS_2 nanostructure from Fig.2**a** was synthesized using chemical vapor deposition on a TEM grid. The Si TEM grid has nine viewing windows with a Si_3N_4 thin film spanning across the entire grid (EMS Catalog #76042). A small amount of MoO_2 (99 %, Sigma-Aldrich 234761) was drop-casted onto the TEM grid from a solution of 2.6 *mg* of MoO_2 suspended in 6 *ml* of isopropanol. Next, the grid was placed in an alumina crucible at 2 *cm* from 7.5 *mg* of MoO_2 powder. The crucible was placed in the middle of a gradient tube furnace from Carbolite Gero, upstream from another alumina crucible containing 400 *mg* of sulfur (99.5 %, Alfa Aesar 10785). Argon was used as a carrier gas with a flow rate of 100 *sccm*. The middle zone was heated to 780 °C, and the sulfur reached a maximum temperature of 270 °C.

The MoO_3 nanorod from Fig.6**a** was synthesized using similar conditions to the MoS_2 nanostructure. In this case, the TEM grid was placed at a distance of 1.2 *cm* from 24.4 *mg* of MoO_2 powder. Furthermore, 50 *mg* of WO_3 powder (99.9%, Sigma-Aldrich 95410) was placed next to the MoO_2 powder.

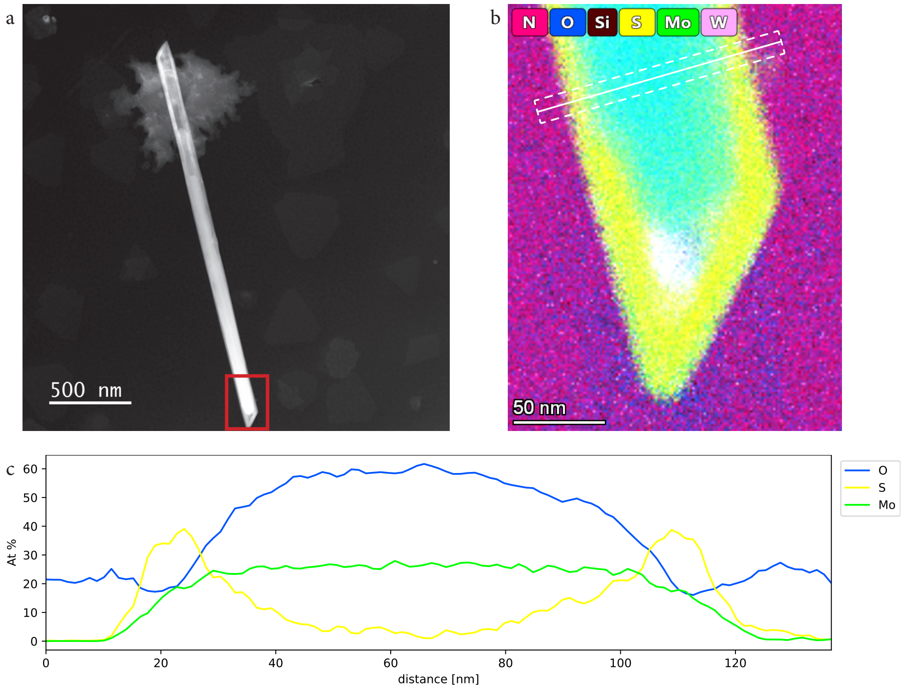

Figure S3: a) Low magnification high-angle annular diffraction (HAADF) image of a representative MoO_3 nanorod, synthesized using the CVD method detailed above. **b)** An energy dispersive X-ray (EDX) color map of the tip of the MoO_3, indicated by the red box in **a**. **c)** An EDX linescan across the width of the MoO_3 nanorod, indicated by the white line in **b**. The linescan shows the atomic percent (At%) for oxygen (blue), sulfur (yellow), and molybdenum (green). The MoS_2 /MoO_3 core-shell nature of the nanorod is clearly visible.

S4 Virtual annular dark field image

Fig. S4 highlights how a virtual ADF detector is created by applying an annular mask to each NBED pattern (pixel).

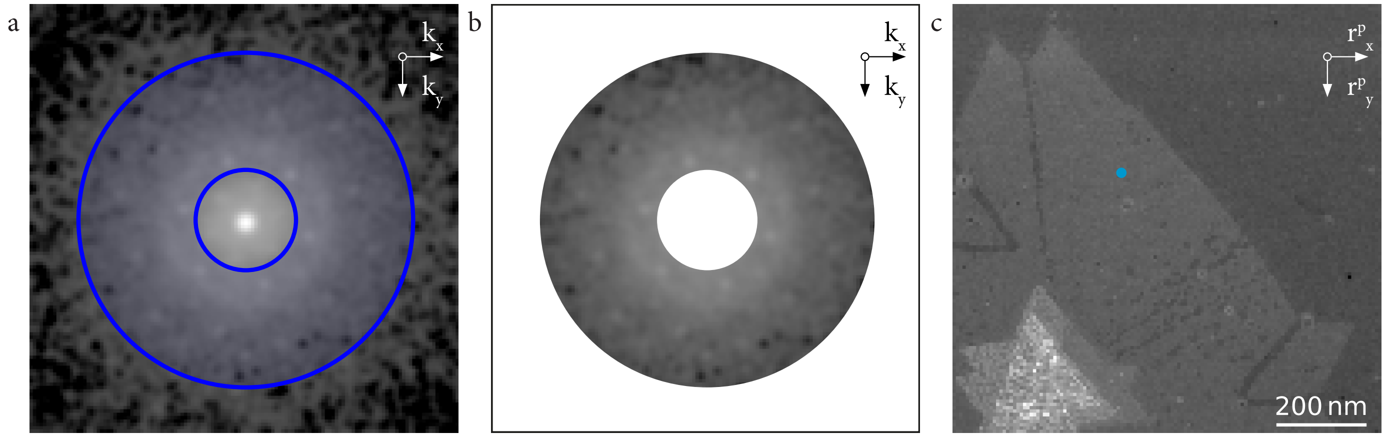

Figure S4: a) A virtual ADF detector is created by applying an annular mask to each NBED pattern (pixel). **b)** The electron intensity is integrated over the annular mask giving a total electron count for each pixel. **c)** The process in **a** and **b** is repeated with the same annular mask for each NBED pattern in the 4D dataset, resulting in the virtual ADF image. The blue dot in the ADF image indicates the location of the NBED pattern in **a**.

S5 Difference of Gaussian method

Fig. S5 describes how the difference of Gaussian (DoG) method is used to accurately detect peaks in an EWPC pattern. The top row of images represents EWPC patterns of the MoO_3 nanorod shown in Fig.6**a**. The EWPC in Fig. S5**a** is the unfiltered EWPC with the intensity profile along the blue linescan in Fig. S5**e**. The EWPC in Fig. S5**b** has a small Gaussian filter applied to it. In the corresponding linescan in Fig. S5**f** shows that this small Gaussian filter hardly changes the EWPC pattern, as we can still see small variations in intensity. Fig. S5**c** shows the same EWPC pattern with a large Gaussian filter applied to it. The corresponding linescan, we can clearly see that all the small intensity variations are filtered out. However, this increases the risk of filtering out small features.

The EWPC in Fig. S5**d** is computed by taking the difference between Fig. S5**b** and Fig. S5**c**. This EWPC pattern, with the DoG applied, has only the sharp points remaining and almost no noise around the center beam. The corresponding linescan in Fig. S5**h** shows 5 distinct peaks and a much lower noise floor. This DoG-filtered EWPC is, therefore, ideal for automatically tracking the position of the EWPC peaks by looking for the local maximum, justifying the approach taken in our work.

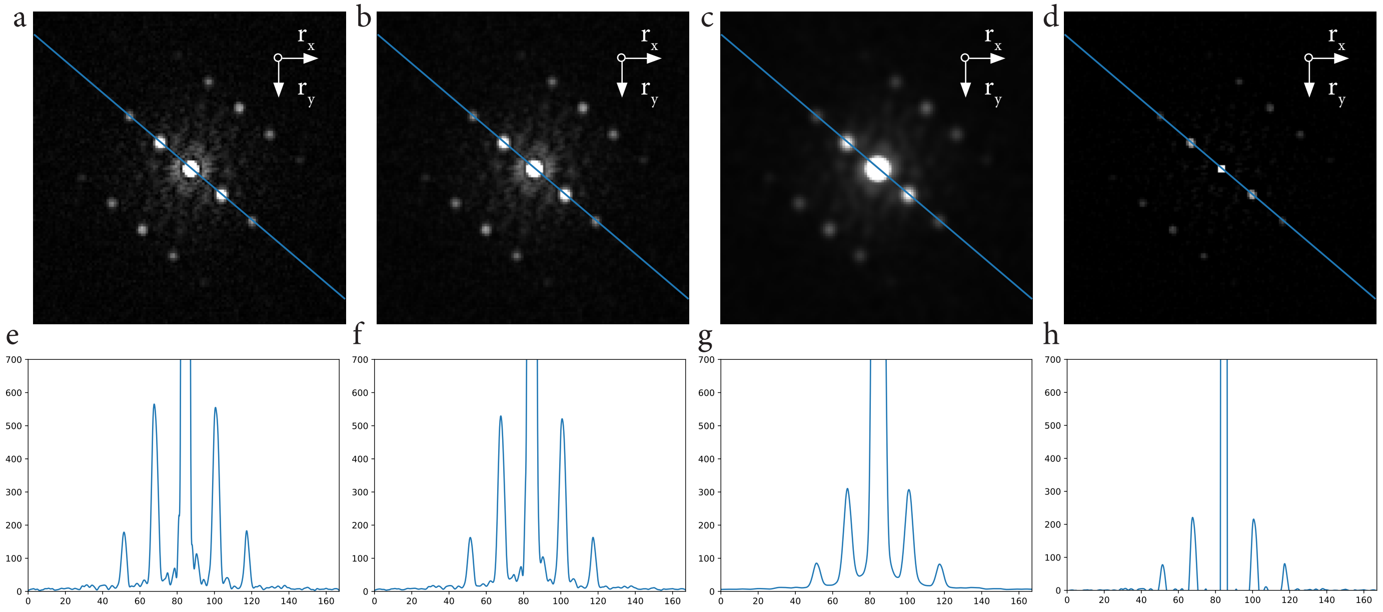

Figure S5: a-d) The EWPC pattern of the MoO_3 nanorod from Fig.6**a**. **a** is unfiltered and **b-d** are blurred with successively stronger Gaussian kernels. **e-h)** The linescan of the EWPC patterns indicated in **a-d.**

S6 Study on the systematic errors in the StrainMAPPER method

To validate the systematic error in the StrainMAPPER method, one needs a well-known reference sample or a dummy dataset in which the expected strain values are known in advance. To facilitate the benchmarking of the systematic error in our method, we have included the ability to generate dummy datasets in the StrainMAPPER package. This section discusses various dummy datasets that demonstrate our method's systematic error. Furthermore, we demonstrate that our method achieves greater accuracy than the difference of the Gaussian (DoG) method for real-world measurements by combining the DoG peak track with the center-of-mass (CoM) method to determine the center of EWPC with sub-pixel precision.

The dummy dataset used for validation consists of the same EWPC pattern for each real space pixel. The resulting strain and rotation across the entire "specimen" should be zero when there are no systematic errors. As a result, any deviation from zero strain is attributed to systematic errors in tracking the EWPC peaks. First, we track the EWPC peak positions for an ideal EWPC pattern, where the peaks are precisely located at the center of a detector pixel, and no noise is present in the EWPC pattern. The DoG peak tracking method accurately identifies the EWPC peak locations for each real space pixel, as evidenced by the peak cluster shown in Fig. S6.1**a**. This figure illustrates the flawless EWPC pattern and six identical EWPC peak clusters, each containing 16,384 counts (128x128). The resulting strain map will display zero strain across the entire "specimen" due to the uniformity of the EWPC pixel location within each cluster. It's worth noting that the increased precision offered by the CoM method doesn't provide any advantage in this scenario, as the centers of the EWPC peaks are already precisely positioned on individual detector pixels.

Subsequently, we introduce a 30-degree rotation to the EWPC pattern, causing the center of the EWPC peaks to no longer align perfectly with a single detecor pixel. As a result of this rotation, interpolation of pixel intensities becomes necessary because a misalignment between the EWPC peak center and the pixel center is introduced. This scenario more closely resembles a real-world EWPC pattern. Due to the limited pixel density of 4D-STEM detectors, such as the EMPAD, it is highly likely that the center of an EWPC peak spans more than one pixel. Furthermore, we introduce random noise to each EWPC pattern, creating a unique EWPC pattern for each real space pixel in the 4D-dataset .

To match the noise level of the dummy dataset with the noise level of an actual 4D-STEM measurement, we calculate the mean and maximum intensity, standard deviation, and the noise quality (NQ) for each EWPC pattern and then average these values over the entire 4D dataset (refer to Table S1).

Here, the noise quality is given by the equation:

$$\mathrm{NQ} = \frac{\sqrt{Mdn(\epsilon_{xy}^2)}}{\max(\epsilon_{xy})} \tag{18}$$

Fig. S6.1**b** shows a three-dimensional (3D) plot of one of the EWPC patterns in the 4D dataset

Table S1: This table presents the statistical analysis results, including mean intensity, maximum intensity, standard deviation, and noise quality, for each EWPC pattern within the 4D dataset. The values are computed over the entire dataset, providing insights into the noise characteristics of the patterns, which closely resemble real-world scenarios.

	4D-STEM Data	Dummy Data
Mean	37.75	34.42
Max	372.4	375.0
StDev	28.27	27.83
NQ	9.054E-2	9.12E-2

of the MoS_2 nanostructure from Figures 2-4. Fig. S6.1**c** presents a 3D plot of one of the generated EWPC patterns from the dummy dataset. The mean and maximum intensity, standard deviation, and the noise quality for this dummy dataset are listed in Table S1. Comparing Figure S6.1**b** and S6.1**c** alongside the values in Table S1, one can observe that we can introduce a similar noise level to the dummy dataset as the one present in the actual dataset.

The results of the DoG peak track on the dummy dataset are displayed in Fig. S6.1**d**. One can observe 15 unique peak locations. The pixel interpolation, introduced by the 30-degree rotation, combined with the random noise, causes the DoG method to identify the center or the EWPC peaks in several locations. One can observe this effect most clearly in the map of the x component of the vector belonging to cluster 1 $(V1_x)$ in Fig. S6.1**e**. This figure shows that the EWPC peak position, as determined by the DoG method, can vary by 1 pixel. This integer pixel precision adversely affects the accuracy of the strain measurement, resulting in strain values ranging from +0.8% to -8.1%, with a standard deviation (σ) of +2.7%, as depicted in Fig. S6.1**f**.

Improving the accuracy of the EWPC peak center location can enhance the precision of the strain measurements. The CoM method implemented in StrainMAPPER utilizes the result of the DoG peak track as the center of a small mask. The center of this masked area's weighted intensity, or CoM, determines the EWPC peak center with sub-pixel accuracy. The vector map in Fig. S6.1**g** exhibits a much finer granularity in the x coordinate of the EWPC peak position compared to the binary vector map in Fig. S6.1**f**. The sub-pixel precision in the EWPC location results in a more accurate strain map (ϵ_{xx}) with values ranging from +1.6% to -4.5% and $\sigma = 1.2\%$, as shown in Fig. S6.1**h**). The size of the CoM mask determines the accuracy of the strain measurement. When the mask is too small, there is no significant benefit to using the CoM method. Conversely, when the mask is too large, the noise in the EWPC pattern starts to influence the position of the CoM of the EWPC peak. The graph in Fig. S6.1**i** illustrates the maximum and minimum strain values (ϵ_{xx}) and the standard deviation for various CoM mask radii (r), where $r = 0$ corresponds to just using the DoG method. This graph reveals that the optimal choice lies with a CoM mask radius of 2 pixels for this dataset. The graph in Fig. S6.1**j** presents the mean squared error between the different strain maps $(\epsilon_{xx}, \epsilon_{yy}, \text{and } \epsilon_{xy})$ and the expected maps with no strain $(\epsilon = 0)$, for different CoM mask radii.

Once again, a CoM mask with a radius of 2 pixels proves to be the most accurate. The low pixel density of the 4D-STEM detector can cause the perceived center of the EWPC peak to shift considerably from one EWPC pattern (real-space pixel) to the next. These shifts, in turn, introduce pixelated noise in the strain map, as seen in Figure S6.1**h**. With StrainMAPPER, we can apply a Gaussian blur to the final strain map to further reduce the pixelated noise. The Gaussian filter functions as two sequential 1D-convolution filters [3], mapping the pixel intensity to a weighted average of the surrounding pixels, with the original pixel receiving the highest weight. The Gaussian filter will reduce the noise in the strain map at the expense of spatial resolution and sharpness. However, since StrainMAPPER aims to map strain over large micrometer-sized structures, a slight reduction in spatial resolution in exchange for a less noisy strain map is a favorable tradeoff. The complete set of strain maps, with a Gaussian filter ($\sigma_G = 0.65$) applied, are presented in Figures S6.2**a-d**, with a maximum strain value for ϵ_{xx} of +0.6% and a minimum value of -3.1% with $\sigma = 0.5\%$. The average σ over ϵ_{xx}, ϵ_{yy}, and ϵ_{xy} is 0.36%, resulting in a 3σ confidence value of 1.1%. Therefore, it can be stated that all strain measurements in the main text have an uncertainty of $\pm1.1\%$. The same process can be applied to the rotation map in Figure S6.2**d**, resulting in a standard deviation of $\sigma = 0.16^\circ$ and an uncertainty of $\pm0.47^\circ$.

Finally, we explore a different approach to increase the accuracy of the StrainMAPPER method. Ensuring that the EWPC pattern spans more pixels reduces the impact of a one-pixel shift in the peak location on the strain calculation. This reduction can be achieved by either increasing the pixel density of the 4D-STEM detector or ensuring that the EWPC pattern spans more pixels. The first option is constrained by hardware design, while the second option can be achieved by decreasing the camera length of the TEM [1]. For example, we created a dummy sample where the EWPC peaks are separated from the center by three times as many pixels. The average standard deviation over ϵ_{xx}, ϵ_{yy}, and ϵ_{xy} decreased to $\sigma = 0.26\%$. When applying the Gaussian blur to the strain map, the average standard deviation decreased even further to $\sigma = 0.116\%$. Therefore, when the measurement conditions allow for a well-separated EWPC pattern, the accuracy of the StrainMAPPER method can increase to $\pm0.35\%$ (3σ).

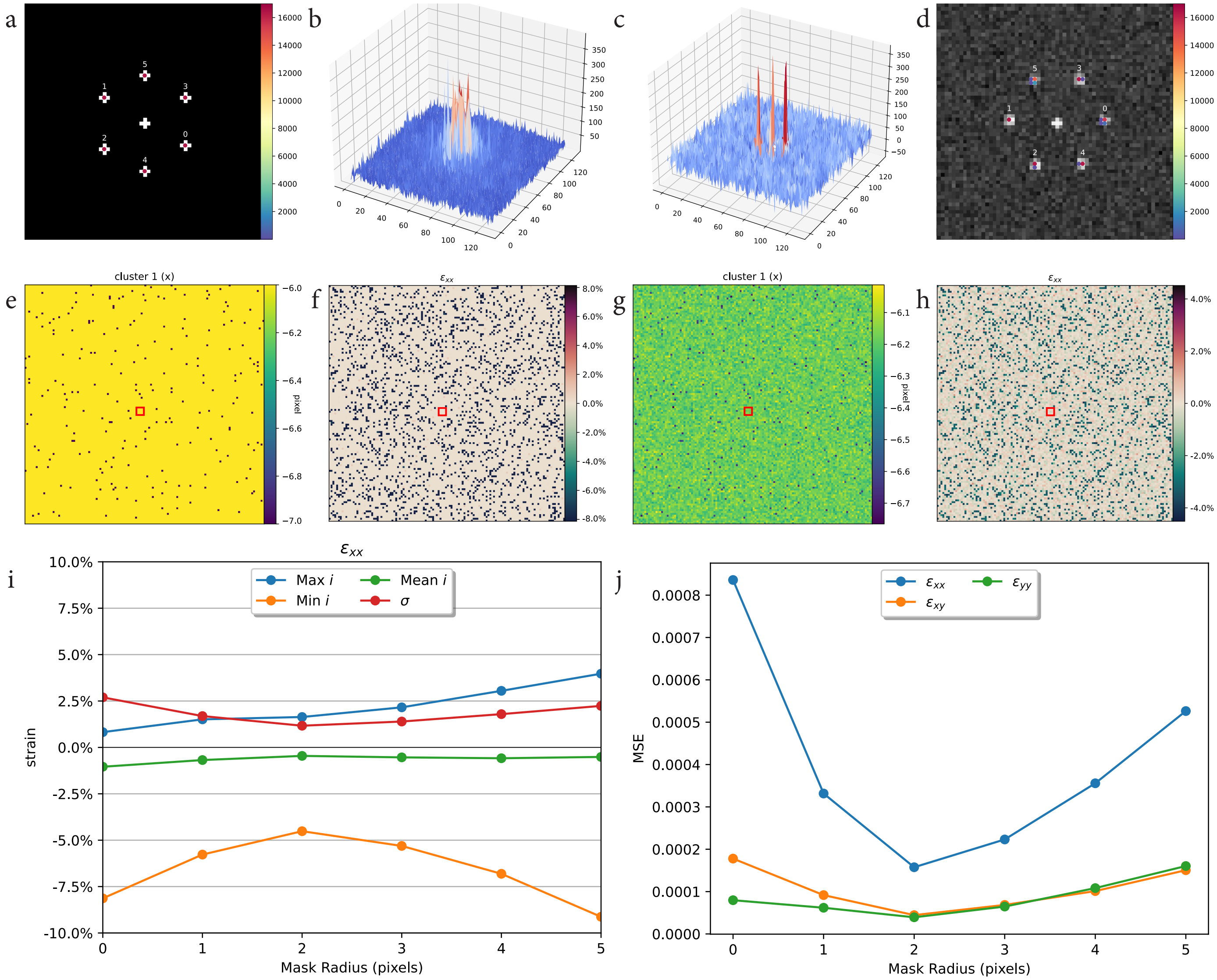

Figure S6.1: This figure illustrates the steps taken to quantify the accuracy in StrainMAPPER strain measurements. **a)** Shows the peak cluster for a perfect 4D dataset without noise. **b-c)** Show 3D plots of a single EWPC pattern from a real-world and dummy dataset, respectively. **d)** Displays the peak cluster of the dummy dataset with noise. **e-f)** The results of the DoG peak tracking method on $(V1_x)$ and the corresponding strain map (ϵ_{xx}). The strain values exhibit variations from +0.8% to -8.1% with $\sigma = +2.7\%$. **g-h)** A vector map showcasing the finer granularity achieved by the CoM method in determining the EWPC peak center and the corresponding strain map. The strain values range from +1.6% to -4.5%, with $\sigma = 1.2\%$. **i)** Shows the Max, Min, and $Mean$ strain values and the standard deviation in ϵ_{xx} for different mask radii. **j)** Shows the MSE for different mask radii. Both **i** and **j** show an optimum at a mask size of 2 pixels.

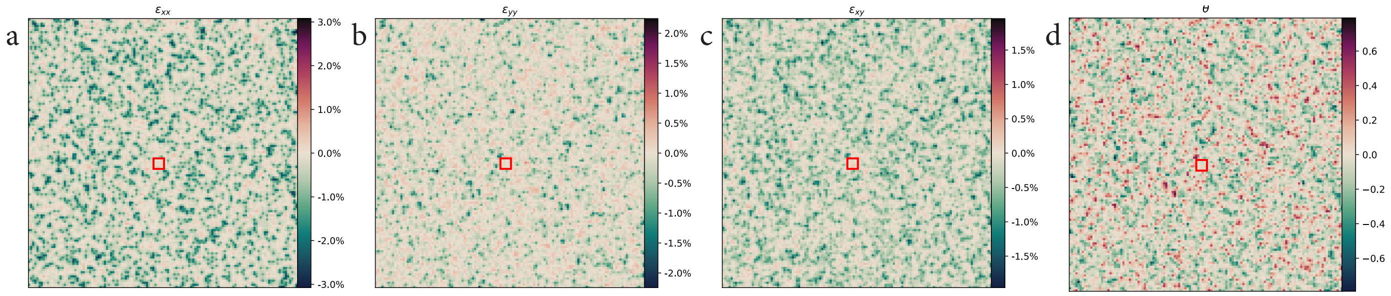

Figure S6.2: a-d) Showcase the complete set of strain maps after applying a Gaussian filter with a standard deviation (σ_G) of 0.65. This post-processing step effectively reduces pixelated noise in the strain maps, improving visual clarity and accuracy. The strain map (ϵ_{xx}) exhibits variations from +0.6% to -3.1% with $\sigma = 0.5\%$

S7 Twisted Heterostructures: $MoSe_2$/WSe_2 Study

Twisted heterostructures formed by stacking two-dimensional (2D) crystals have gained attention due to their unique electronic and optical properties. One of the most intriguing phenomena in such materials is the emergence of Moiré patterns, which arise from the relative misalignment or ”twist” between adjacent layers. These patterns, in turn, are closely tied to local strain fields within individuals layers. In this section, we not only delve into the detailed strain maps of the heterostructure but also elucidate the underlying mechanisms that differentiate the Moiré twist angle (α) between adjacent layers from the rigid rotation deformation within the crystal lattice of a single layer. For this purposes, we use a multilayer stack of $MoSe_2$ and WSe_2 flakes misaligned by approximately 15 degrees.

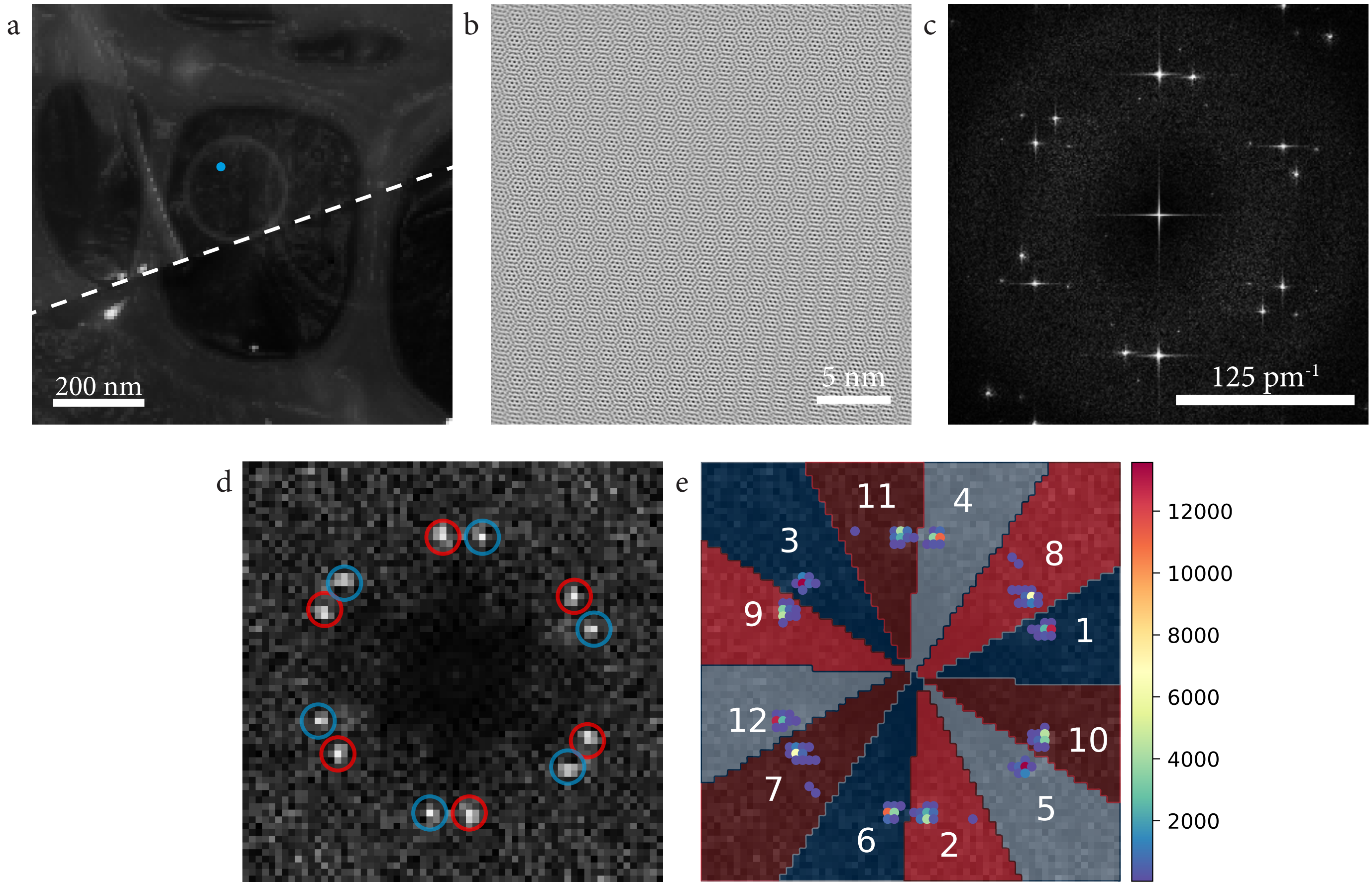

Figure S7.1: a) An ADF image from the $MoSe_2$/WSe_2 multilayer. The white line indicates the border between the single-layer and double-layered area. **b)** A filtered High-Resolution TEM image of the Moiré lattice. **c)** The fast Fourier transform (FFT) of the HR-TEM image in **b**. **d)** The EWPC taken from the position marked by the blue dot in **a**. Similar to the FFT in **c**, the EWPC contains two hexagonal patterns, showcasing the Moiré lattice. **e)** The cluster plot showcases the position of all the EWPC peaks appearing at least 50 times. The 12 clusters represent the different layers within the multilayer stack, each denoted by either blue or red clusters.

Fig. S7.1**a** presents an Annular Dark-Field (ADF) image obtained from a 4D-STEM dataset of the multilayered stack. This image reveals the overall morphology of the stack, highlighting the presence of multiple layers. Fig. S7.1**b** showcases a high-resolution TEM (HR-TEM) image of the Moiré lattice, emphasizing the fine details within the crystal structure. In Figs. S7.1**c**, we display the Fast Fourier Transform (FFT) of the Moiré lattice, demonstrating the two sets of the characteristic hexagonal diffraction pattern of $MoSe_2$ and WSe_2, which indicates the presence of a Moiré superlattice. Fig . S7.1**d** presents an EWPC pattern extracted from the 4D dataset at the location marked by the blue dot in Fig. S7.1**a**. This EWPC pattern also exhibits the same hexagonal Moiré pattern observed in the FFT, affirming that we can probe the Moiré lattice with nanometer precision even when imaging a micrometer-sized structure, as depicted in Fig. S7.1**a**. Fig. S7.1**e** illustrates a cluster plot of all the EWPC peak positions, revealing 12 distinct clusters. These clusters correspond to the different layers within the multilayer stack, with each layer's peaks denoted by either blue or red clusters.

By selecting two clusters that belong to the same layer, we can map the local strain fields and the rigid rotation (θ) within a single layer. In Figs. S7.2**a-d**, we display strain and rotation maps for one of the layers in the multilayer stack, generated using clusters 1 and 4 from the cluster plot in Fig. S7.1**e**. These maps reveal considerable tensile strain at areas where the multilayer hangs over the holes in the lacy-carbon TEM grid. In contrast, Figs. S7.2**e-f** present strain and rotation maps for the second layer in the multilayer stack, utilizing clusters 8 and 11 from the cluster plot in Fig. S7.1**e**. The rotation map (Fig. S7.2**f**) highlights a slight, clockwise rotation with respect to the reference area (indicated by the red box). This rotation arises from the ripple within this layer and represents an excellent example of strain-induced rigid rotation deformation within a single layer.

The schematic in Fig. S7.3**a** introduces the concept of a twist angle (α) between two separate layers within a multilayer stack. This twist angle arises from the relative rotation of the crystal lattices in adjacent layers, contributing to the formation of Moiré patterns. On the other hand, Fig. S7.3**b**illustrates the concept of rigid rotation deformation within a single layer. The rotation angle (θ) denotes the rotation of the crystal lattice between the measured location and a reference area. We illustrate this concept with the rotation of the EWPC pattern in this figure. Local variations in strain or defects can drive such rotations within individual layers.

Finally, in Fig. S7.3**c**, we present a map showing the twist angle (α) between two layers within the multilayer stack. One can determine the twist angle by measuring the angle between the EWPC peaks from two neighboring clusters: one from a red cluster and one from a blue cluster. The twist angle in Fig. S7.3**c** denotes the angle between the EWPC peaks in clusters 11 and 4. Notably, the twist angle varies across the stack. On the right side of the fold, the twist angle is approximately 15 degrees, consistent with the measurement from the FFT in Fig S7.1**c**. However, on the left side of the fold, a smaller twist angle is observed due to the presence of a slight clockwise rotation in one of the layers, as demonstrated in Fig S7.2**f**. In summary, our method not only enables us to map strain within multilayered materials, but also allows us to distinguish between the twist (α) in Moiré lattices and strain-induced rigid rotation deformations (θ).

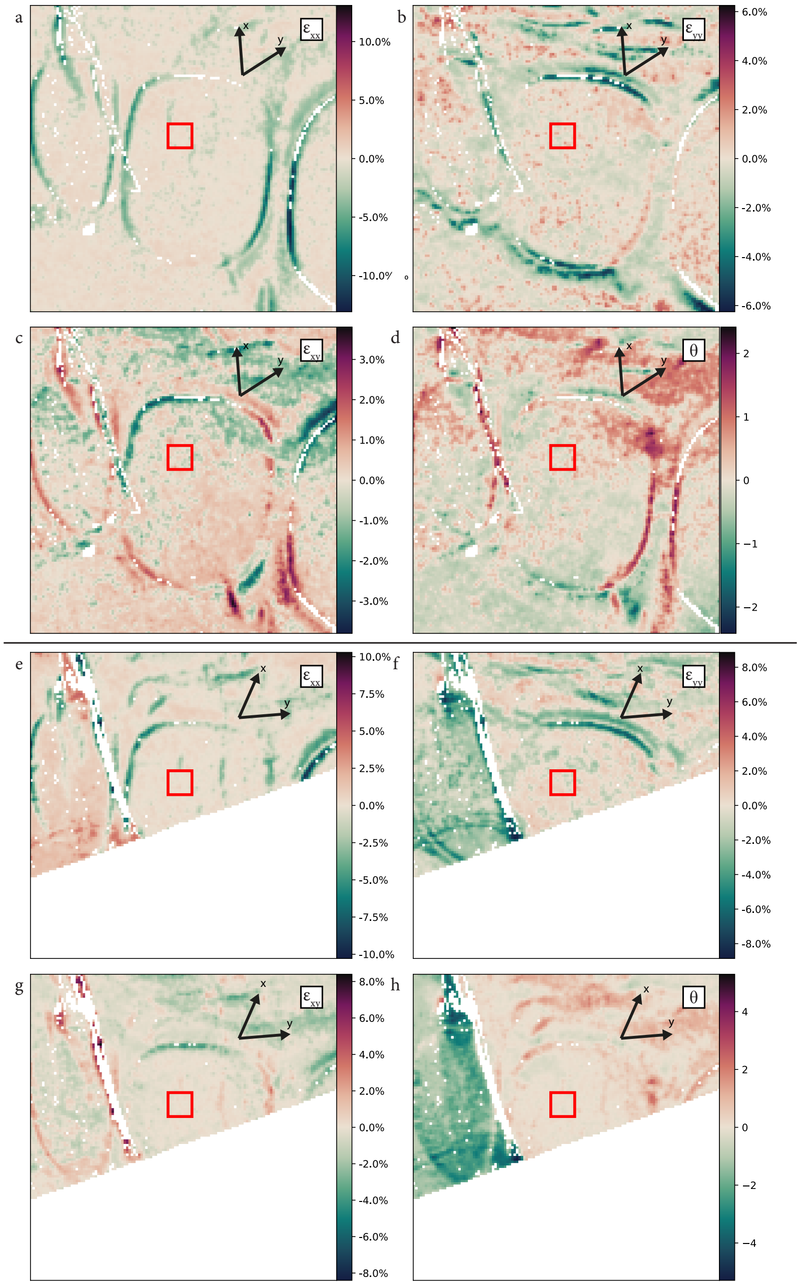

Figure S7.2: a-d) Strain and rigid rotation deformation maps of one of the layers in the multilayer stack. The red box indicates the reference area. One can observe considerable compressive strain at the edges of holes in the lacy-carbon TEM grid. **e-f)** The strain and rigid rotation deformation maps of the second layer in the multilayer stack. This second layer is visible only in the top half of the map. Analogous to **a-d**, there is noticeable compressive strain around the edges of the holes in the lacy-carbon TEM grid and around the fold spanning the width of this layer. Additionally, there is a relative rigid rotation deformation ($\theta = 2^{\circ}$) between the area on the left side of the fold and the reference area, which is located on the right side of the fold.

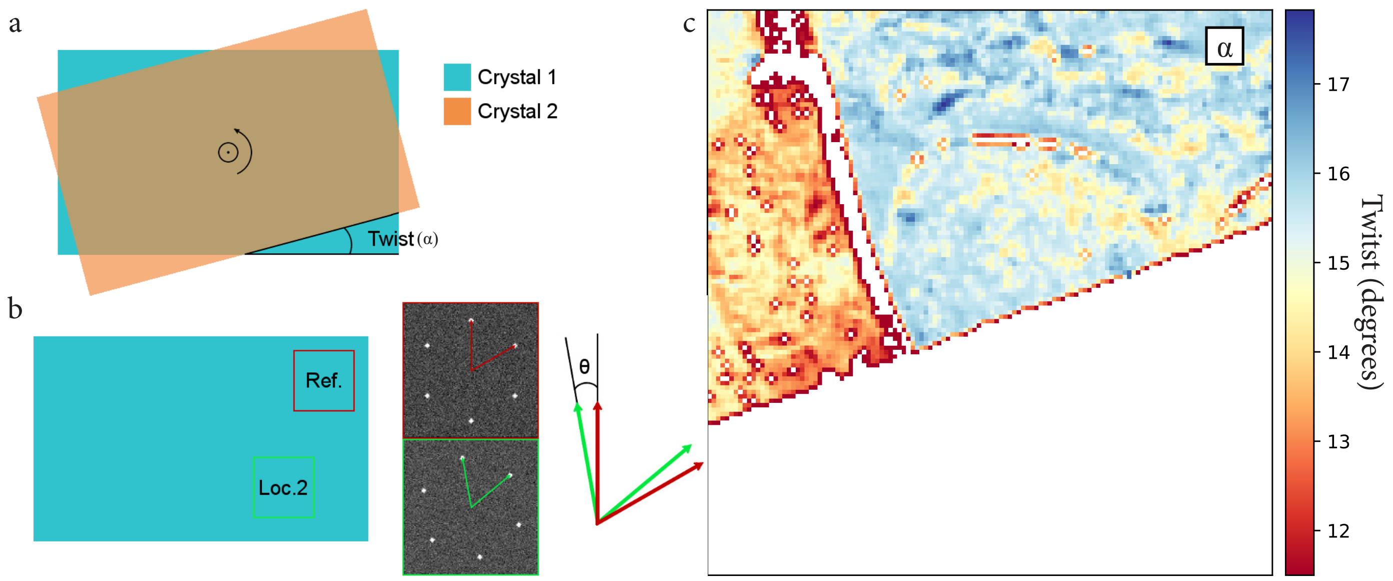

Figure S7.3: a) A schematic illustrating the Moiré twist angle (α) between two layers in a multilayer stack. **b)** A representation of the rigid rotation deformation (θ) within the crystal lattice of a single layer. This deformation is defined as the relative rotation of the lattice in the measured location compared to that in a reference area, as depicted by the rotation of the EWPC patterns. **c)** A map highlighting the Moiré twist angle (α) between the two layers in the stack. A slight variation in the twist angle is observed between the left side $(\alpha \simeq 13^{\circ})$ and the right side $(\alpha \simeq 15^{\circ})$ of the multilayer

S8 Strain and rotation maps of the MoS_2 Moiré multilayer

Fig. S8 displays the complete set of train and rotation maps of the MoS_2 Moiré multilayer specimen.

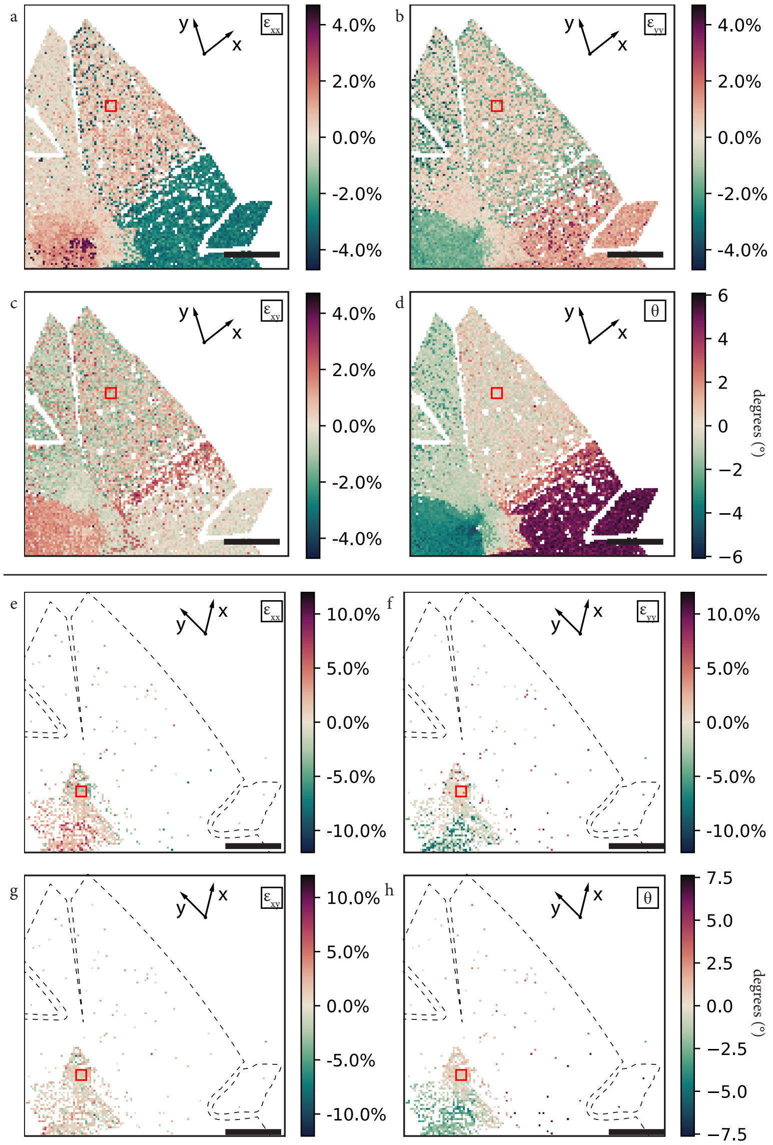

Figure S8: a-d) The strain and rotation maps of the first MoS_2 layer in the Moiré point cloud in Fig.5b. These maps were calculated using the EWPC peaks from cluster 4 and cluster 2. The region used as a reference is denoted by the red box. These strain maps are almost identical to the strain and rotation maps in Fig.4, indicating the flexibility of our approach. It doesn't matter whether we track the EWPC peaks of the Moiré pattern separately or all at once. We can determine the strain in both layers with similar accuracy. **e-h)** The strain and rotation maps of the second MoS_2 layer in the Moiré point cloud in Fig.5b. These maps were calculated using the EWPC peaks from cluster 9 and cluster 5. The reference point is denoted by the red box. The scale bars in all figures are 200 nm.

S9 Strain and rotation maps of the MoS_2/MoO_3 heterostructure

Fig. S9.1 displays the full set of strain and rotation maps of the MoO_3 nanorod and Fig. S9.2 displays the full set of strain and rotation maps of the MoS_2 thin film.

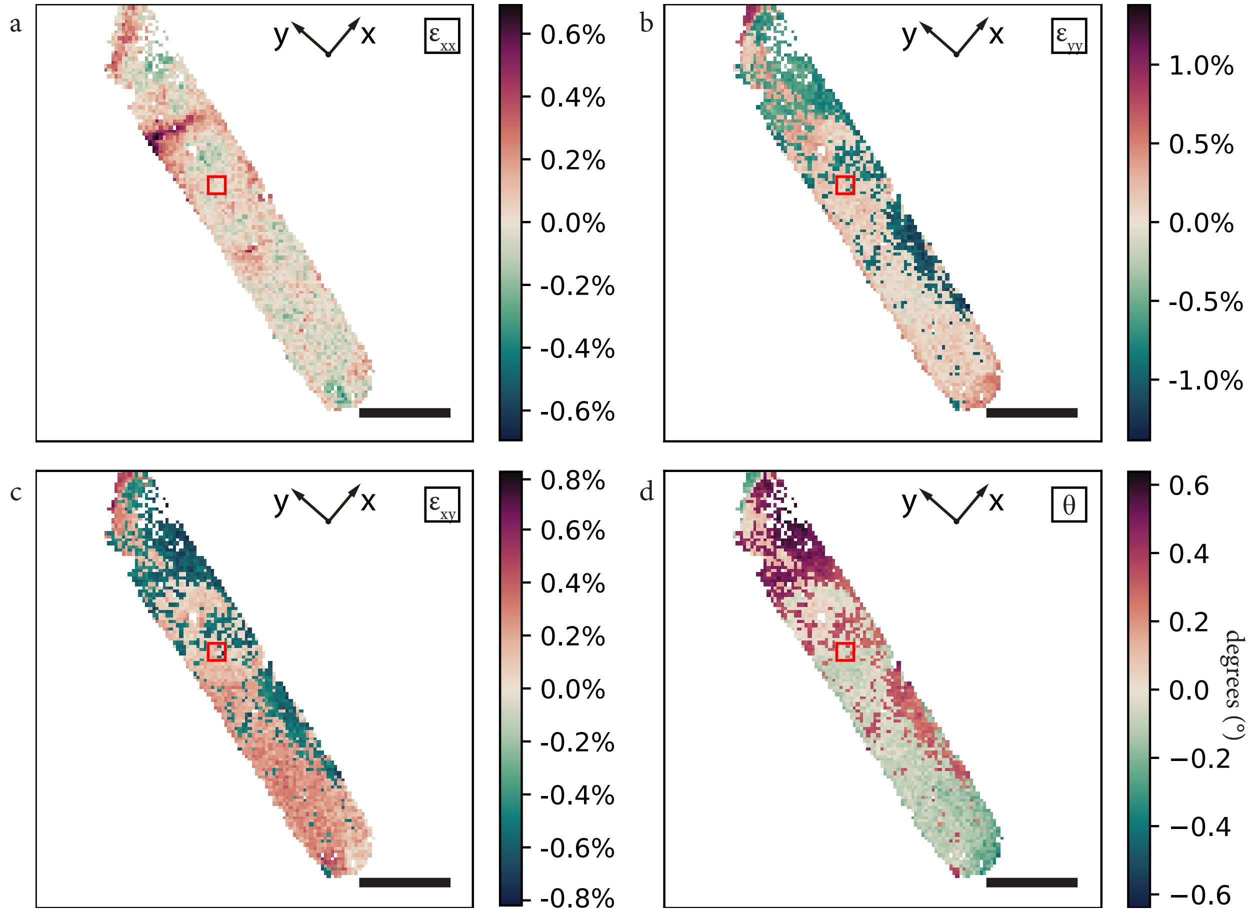

Figure S9.1: a-d) The complete strain and rotation maps of the MoO_3 nanorod. These maps were calculated using the EWPC peaks from cluster 4 and cluster 9 in the point cloud from Fig.6**d**. The region used as a reference is denoted by the red box. The scale bars in all figures are 50 nm.

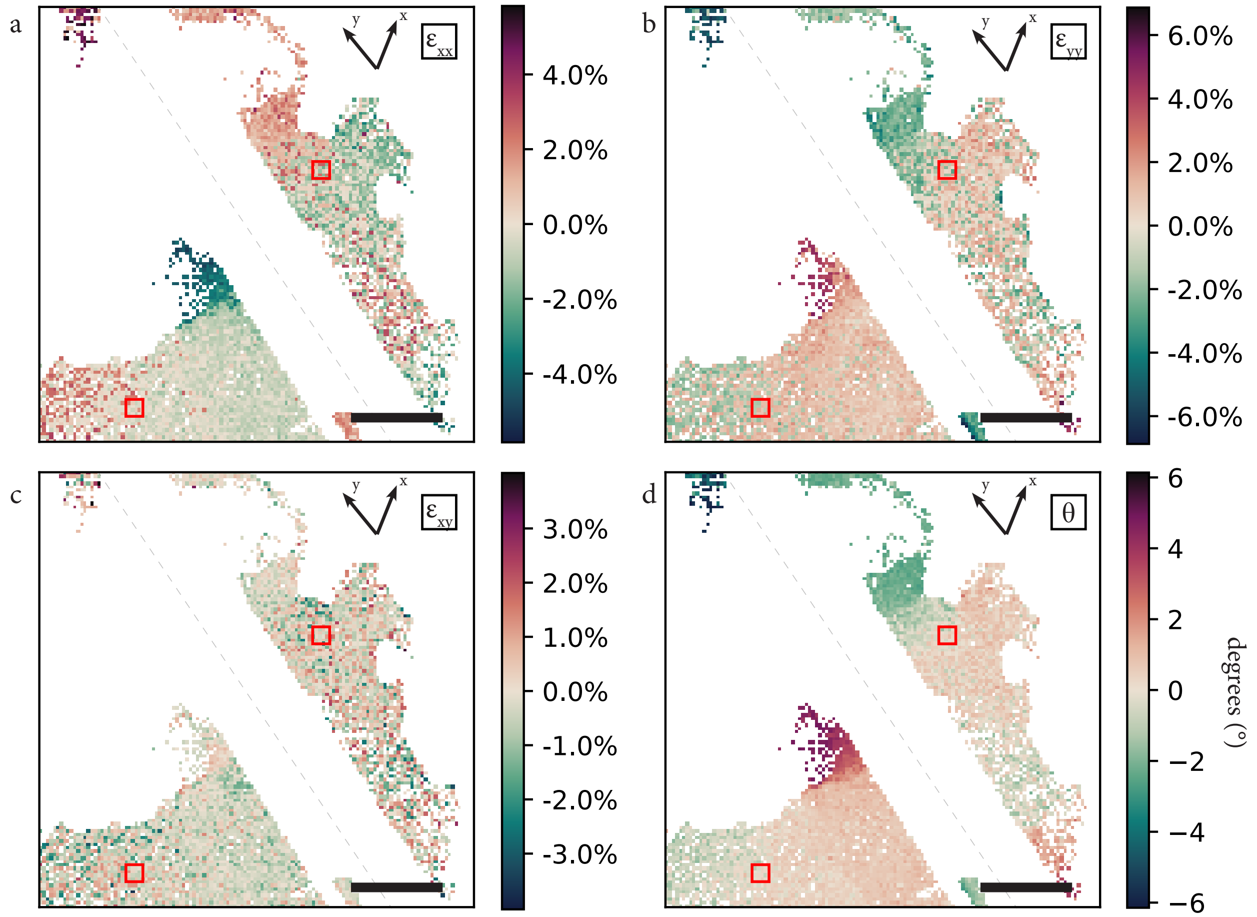

Figure S9.2: a-b) The strain and rotation maps of the MoS_2 thin film. These maps were calculated using the EWPC peaks from cluster 5 and cluster 2 in the point cloud from Fig.6d. The rotation map in Fig.6e shows that the MoS_2 thin films on the left and the right are separated by the MoO_3 nanorod. Therefore, two reference areas (indicated by the red boxes) were selected for the strain and rotation maps of the MoS_2 thin films. One for the left and one for the right MoS_2 thin film. The scale bars in all figures are 50 nm.

S10 4D-STEM acquisition details

Table S2 contains the acquisition parameters used in each of the figures shown in the main text.

	Figure 1	Figure 2-5	Figure 6
Accelerating voltage	300 kV	300 kV	300 kV
Convergence semi-angle (mrad)	2.63	0.53	0.53
Camera length (mm)	285	460	285
Dwell time (ms)	1	1	1
Magnification	40K	40K	160K

Table S2: 4D-STEM acquisition parameters used in each of the figures shown in the main manuscript.

S11 Comparison between 4D-STEM strain mapping methods

In this section, we compare the results of the StrainMAPPER method to others aimed at measuring strain fields using 4D-STEM. A direct comparison between StrainMAPPER and different methods, such as py4DSTEM [2], pyxem, and PC-STEM, is not straightforward because each method is developed and optimized for different types of 4D-STEM datasets. Firstly, the py4DSTEM and pyxem methods determine the position of Bragg disks to assess the specimen's strain, while the PC-STEM and StrainMAPPER methods use the position of EWPC peaks to determine the specimen's strain. The optimal TEM acquisition conditions for obtaining clear Bragg disks and distinct EWPC patterns can vary significantly. Therefore, using the same dataset with each method is sometimes unfeasible.

Here, we will compare the strain maps of two specimens using the py4DSTEM (CBED), PC-STEM (EWPC), and StrainMAPPER (EWPC) methods. The first specimen is the same MoS_2 nanostructure as discussed in the main text. Fig. S11.1**a** shows an ADF image of the MoS_2 nanostructure generated by the py4DSTEM method. For the py4DSTEM method, we use a dataset with a large camera length $L = 910mm$ to clearly visualize the Bragg disks. Fig. S11.1**b** shows a CBED pattern taken in the multilayer region of the MoS_2 nanostructure, indicated by the orange dot in Fig. S11.1**a**. The py4DSTEM method can accurately determine the position of multiple Bragg disks in this CBED pattern. However, the diffraction contrast drastically reduces for the thinnest part of the MoS_2 specimen. The CBED pattern in Fig. S11.1**c** shows a CBED pattern taken from the position indicated by the green dot in Fig. S11.1**a**. The very low diffraction contrast of the monolayer MoS_2, combined with the signal from the amorphous Si_3N_4 substrate, causes the py4DSTEM method to lose track of the position of most Bragg disks. The CBED-based methods for determining the strain are less suitable for specimens like those presented here, with a low diffraction contrast and a strong background signal.

The EWPC-based methods provide a way forward for these challenging specimens. For the PC-STEM and StrainMAPPER methods, we use the dataset mentioned in the main text with a small camera length $(L = 460mm)$ for optimal EWPC [1]. Fig. S11.1**d** shows an EWPC generated using the PC-STEM method and the search windows for two EWPC peaks. We have chosen the same two EWPC peaks as in the main text. The resulting strain map (ϵ_{xx}) of the PC-STEM method is shown in Fig. S11.1**e**. The PC-STEM method identifies the local maximum inside the search window to determine the EWPC location with sub-pixel accuracy. This approach becomes problematic for specimens like the one used here, where only certain areas of the 4D dataset contain crystalline material. The PC-STEM method interprets the background signal from the amorphous Si_3N_4 as an EWPC peak, resulting in a strain map with very large strain values even though no crystalline specimen is present at that location.

On the contrary, this problem does not affect the StrainMAPPER method because it uses a two-step approach. First, the DoG peak track method correctly identifies the difference between the EWPC peaks from the crystalline specimen and the amorphous background. Next, the CoM method determines the EWPC peak position with sub-pixel accuracy only for the positions with a crystalline

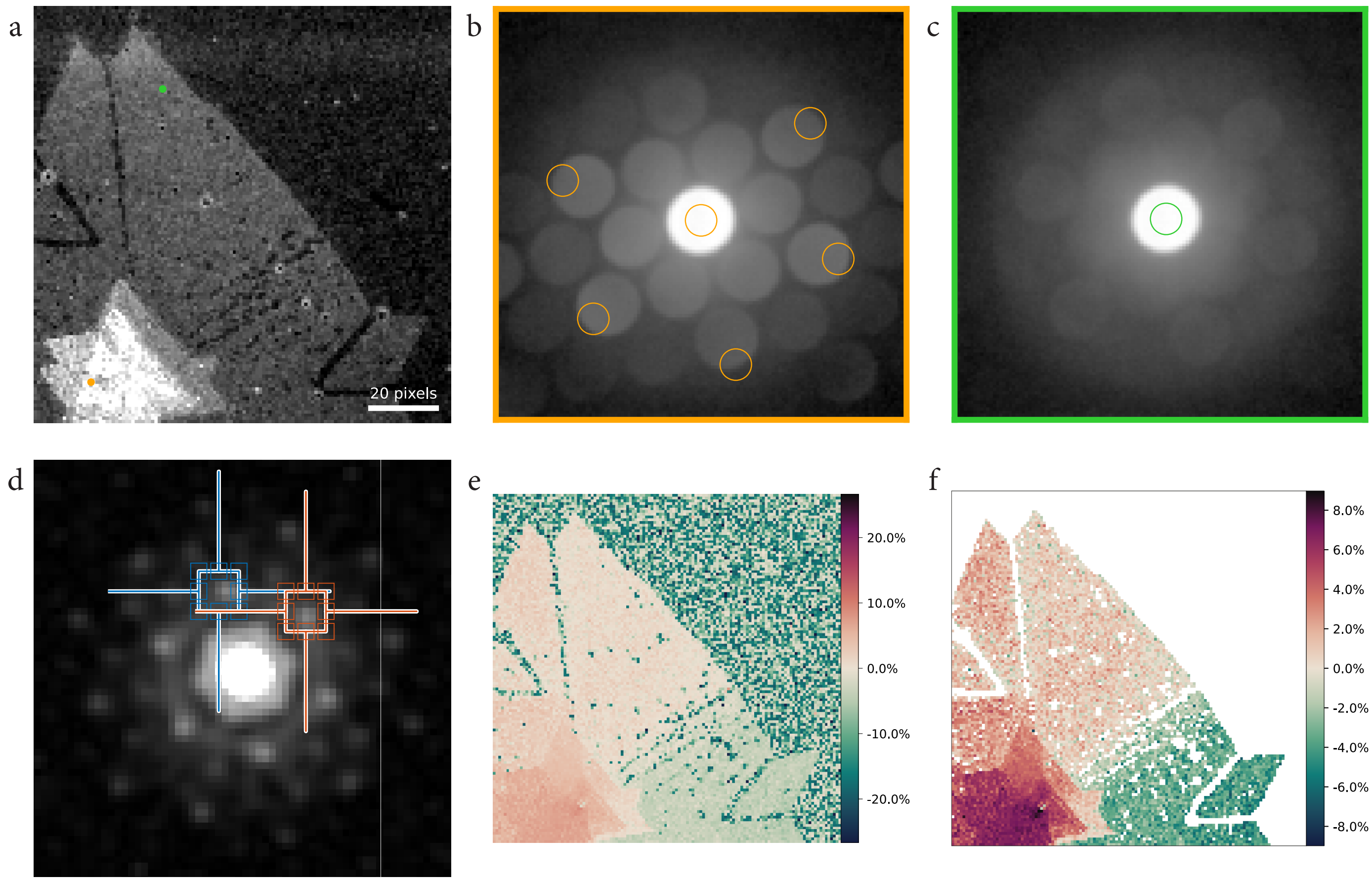

Figure S11.1: a) An ADF image of the MoS_2 nanostructure generated by the py4DSTEM method. **b)** The CBED pattern corresponding to multilayer MoS_2, indicated by the orange dot in **a**. **c)** The CBED pattern corresponding to monolayer MoS_2, indicated by the green dot in **a**. The orange and green circles in **b** and **c** indicate the Bragg disks identified by the py4DSTEM method. **d)** An EWPC pattern generated by the PC-STEM method. The blue and orange search windows indicate the selected EWPC peaks for the strain measurements. **e)** The ϵ_{xx} strain map generated by the PC-STEM method. The method yields large strain values in the substrate due to the amorphous background signal. **f)** The same strain map as in **e**, but with a mask applied to filter out the large "strain" values in the substrate. This strain map is comparable to the one generated by the StrainMAPPER method (see Figure 4 in the main manuscript).

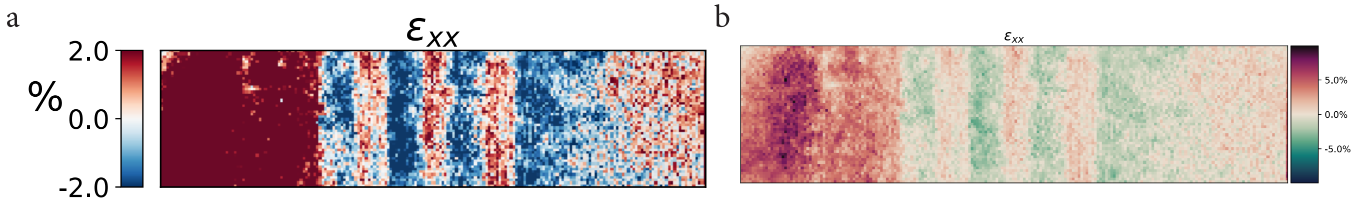

Figure S11.2: **a)** The ϵ_{xx} strain map of the Si/$Si_{0.82}Ge_{0.18}$ multilayer, as represented by the py4DSTEM method, with manually specified minimum and maximum values. **b)** The same strain map as in **a** but now represented in the same style as the StrainMAPPER strain maps, without manually specified minimum and maximum values. The py4DSTEM method is less accurate in resolving the 5 Si/$Si_{0.82}Ge_{0.18}$ multilayers.

specimen present. However, the PC-STEM method accurately measures the strain in the MoS_2 specimen. By applying a post-process mask to the substrate region, we can highlight the strain in the MoS_2 specimen, as shown in Fig. S11.1**f**. It is important to note that PC-STEM currently does not support the use of such a mask. Instead, we used the StrainMAPPER method's results as a mask template.

The final strain map (ϵ_{xx}) generated by PC-STEM method closely resembles the strain map produced by the StrainMAPPER method, as depicted in Fig. 4 in the main text. For comparison with the py4DSTEM, PC-STEM, and StrainMAPPER methods, we utilized a second specimen sourced from the py4DSTEM tutorials Github page (https://github.com/py4dstem/py4DSTEM). The specimen consists of alternating Si/$Si_{0.82}Ge_{0.18}$ multilayers stacked on top of a Si substrate. The py4DSTEM tutorial includes the experimental 4D-STEM dataset along with the tutorial. The acquisition conditions used for this dataset are suitable for generating an EWPC, so we can apply all three methods to the same dataset. Fig. S11.2**a** shows the strain map (ϵ_{xx}) as presented in the py4DSTEM tutorial. To highlight the strain at the SiGe interface, the maximum and minimum values on the scale bar are manually adjusted. Fig. S11.2**b** displays the same strain map without adjusting the scalebar. In this representation, the strain at the SiGe interfaces is no longer discernible due to the large patch on the left. Note that, according to the py4DSTEM tutorial, the accuracy of the obtained strain map is limited due to the thickness of the specimen and the downsampled dataset provided alongside with the tutorial.

The dataset from the py4DSTEM tutorial is well-suited for the EWPC strain mapping techniques used in the StrainMAPPER and PC-STEM methods. In Fig. S11.3**a**, we display the CBED pattern, generated using py4DSTEM, representing the Si substrate, while Fig. S11.3**b** shows the corresponding EWPC, generated by StrainMAPPER. It's worth noting that the EWPC peaks are located only a few pixels from the center, which is not ideal for achieving the highest accuracy with the EWPC methods, as further discussed in section S9. Despite these conditions, it is still possible to track the EWPC peak location along the specimen. Fig. S11.4**a** and Fig. S11.4**b** display the strain maps generated by the StrainMAPPER and PC-STEM methods, respectively. These maps show the strain

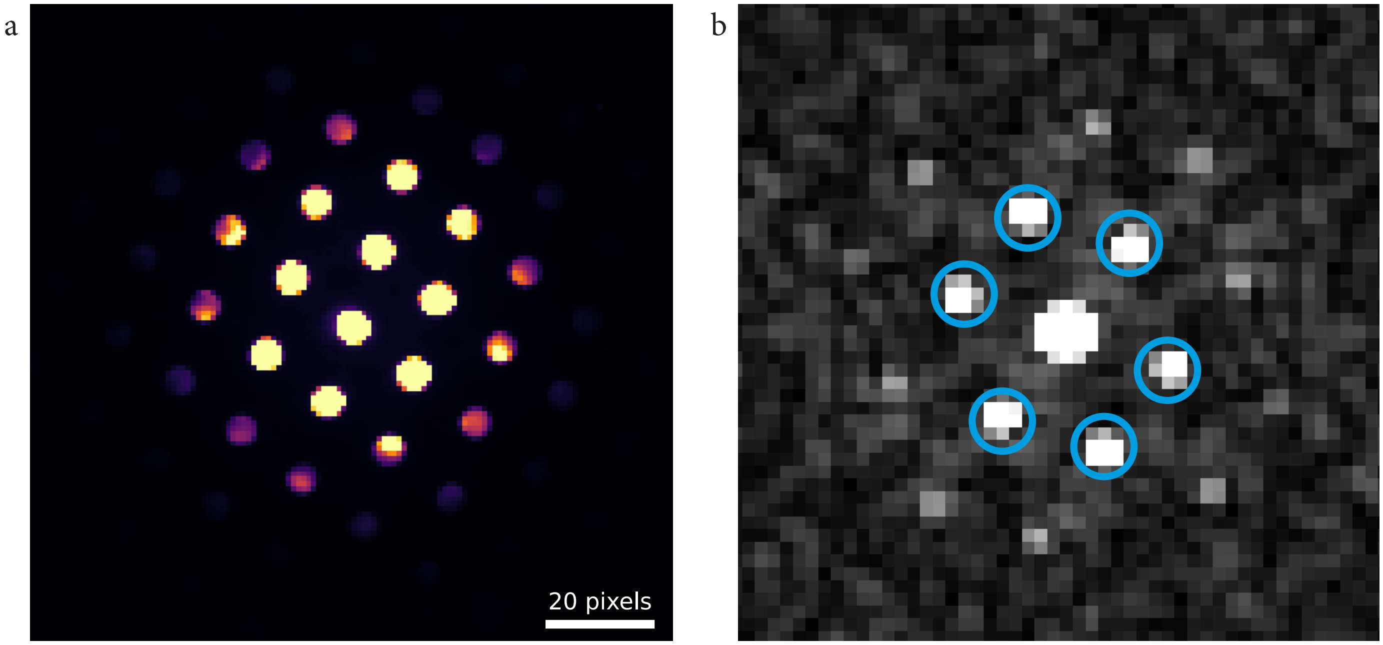

Figure S11.3: **a)** A (mean) CBED pattern of the $Si/Si_{0.82}Ge_{0.18}$ multilayer generated by the py4DSTEM method. **b)** A single EWPC pattern of the multilayer generated by the StrainMAPPER method.

in the x direction (ϵ_{xx}), where the x and y directions are denoted by the Cartesian coordinate system. Both EWPC methods yield similar results and offer improved resolution compared to the py4DSTEM CBED-based method when mapping the 5 $Si/Si_{0.82}Ge_{0.18}$ multilayers. The improved accuracy of the StrainMAPPER method is highlighted in Figs. S11.4**c-d**. Using the py4DSTEM tutorial [2], we can map the average strain along the length of the multilayer and compare it with the estimated strain as calculated with Vengard's law. The StrainMAPPER method can resolve all 5 of the multilayers, and the obtained strain values are closer to the expected ones.

In conclusion, the EWPC-based StrainMAPPER method accurately maps strain, even in specimens with limited diffraction contrast. It effectively distinguishes EWPC peaks from crystalline specimens and background signals generated by thick amorphous layers. Moreover, StrainMAPPER can map strain within SiGe multilayers, even when dealing with thick specimens and downsampled 4D datasets.

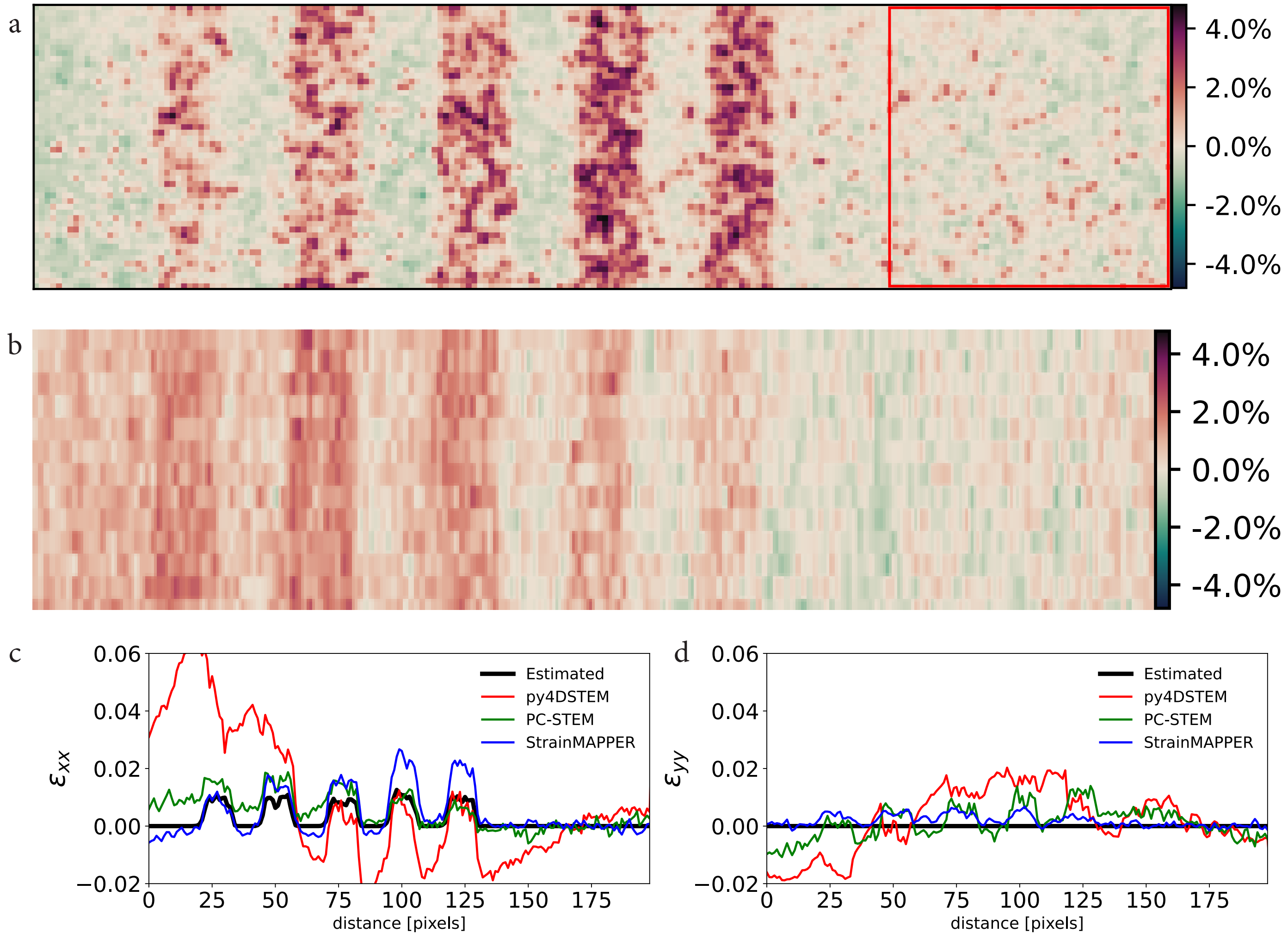

Figure S11.4: a-b) The ϵ_{xx} strain maps, as generated by the StrainMAPPER and PC-STEM methods, respectively. Both methods can resolve all 5 of the Si/$Si_{0.82}Ge_{0.18}$ multilayers. **c-d)** The mean ϵ_{xx} and ϵ_{yy} strain values for all three methods, compared to the estimated strain values by Vengard's law. The strain values of the StrainMAPPER method resemble the estimated strain values the closest.

References

[1] Elliot Padgett et al. "The exit-wave power-cepstrum transform for scanning nanobeam electron diffraction: robust strain mapping at subnanometer resolution and subpicometer precision". In: *Ultramicroscopy* 214 (2020), p. 112994. ISSN: 0304-3991. DOI: https://doi.org/10.1016/j.ultramic.2020.112994. URL: https://www.sciencedirect.com/science/article/pii/S0304399119303377.

[2] Benjamin H Savitzky et al. "py4DSTEM: A Software Package for Four-Dimensional Scanning Transmission Electron Microscopy Data Analysis". In: *Microscopy and Microanalysis* 27.4 (Aug. 2021), pp. 712–743. ISSN: 1431-9276. DOI: 10.1017/S1431927621000477. eprint: https://academic.oup.com/mam/article-pdf/27/4/712/48292107/mam0712.pdf. URL: https://doi.org/10.1017/S1431927621000477.

[3] Pauli Virtanen et al. "SciPy 1.0: Fundamental Algorithms for Scientific Computing in Python". In: *Nature Methods* 17 (2020), pp. 261–272. DOI: 10.1038/s41592-019-0686-2.
